# Dying transplanted neural stem cells mediate survival bystander effects in the injured brain

**DOI:** 10.1038/s41419-023-05698-z

**Published:** 2023-03-01

**Authors:** Wei Han, Eva-Maria Meißner, Stefanie Neunteibl, Madeline Günther, Jörg Kahnt, Amalia Dolga, Cuicui Xie, Nikolaus Plesnila, Changlian Zhu, Klas Blomgren, Carsten Culmsee

**Affiliations:** 1 Department of Women’s and Children’s Health, Karolinska Institutet, Stockholm, Sweden; 2 Henan Key Laboratory of Child Brain Injury, Institute of Neuroscience and Third Affiliated Hospital of Zhengzhou University, Zhengzhou, 450052 China; 3 Institute of Pharmacology and Clinical Pharmacy, University of Marburg, Marburg, Germany; 4 Center for Mind, Brain and Behavior (CMBB), Universities of Marburg and Giessen, Marburg, Germany; 5 Max-Planck-Institute for Terrestrial Microbiology, Department of Ecophysiology, Marburg, Germany; 6 Faculty of Science and Engineering, Molecular Pharmacology – Groningen Research Institute of Pharmacy, Groningen, The Netherlands; 7 Center for Brain Repair and Rehabilitation, Institute of Neuroscience and Physiology, University of Gothenburg, Gothenburg, Sweden; 8 Institute for Stroke and Dementia Research (ISD), University Clinic Munich, Munich, Germany; 9 Pediatric Oncology, Karolinska University Hospital, Stockholm, Sweden

**Keywords:** Cellular neuroscience, Neural stem cells

## Abstract

Neural stem and progenitor cell (NSPC) transplants provide neuroprotection in models of acute brain injury, but the underlying mechanisms are not fully understood. Here, we provide evidence that caspase-dependent apoptotic cell death of NSPCs is required for sending survival signals to the injured brain. The secretome of dying NSPCs contains heat-stable proteins, which protect neurons against glutamate-induced toxicity and trophic factor withdrawal in vitro, and from ischemic brain damage in vivo. Our findings support a new concept suggesting a bystander effect of apoptotic NSPCs, which actively promote neuronal survival through the release of a protective “farewell” secretome. Similar protective effects by the secretome of apoptotic NSPC were also confirmed in human neural progenitor cells and neural stem cells but not in mouse embryonic fibroblasts (MEF) or human dopaminergic neurons, suggesting that the observed effects are cell type specific and exist for neural progenitor/stem cells across species.

## Introduction

The massive loss of neuronal cells observed in neurological diseases has inspired strategies of cell replacement therapies. Transplantation of neural stem and progenitor cells (NSPCs), which possess the ability to self-renew and differentiate into mature neural cells [1], has emerged as a promising therapeutic strategy [2, 3]. Indeed, transplanted NSPCs mediated functional recovery in various models of neurological disease, and cell replacement has been considered one of the main mechanisms responsible for sensorimotor and cognitive improvement after NSPC transplantation [4–6]. While transplanted NSPCs can differentiate into adult neurons [1], the number of such cells integrating into the neural network is relatively small and the majority of NSPCs die soon after transplantation [7–10]. Hence, differentiation may not fully explain the functional improvements observed after transplantation of NSPCs. Therefore, identifying the mechanisms that underlie the therapeutic effects of NSPCs is important from a clinical perspective, for enabling development of standardized therapies for neurodegenerative diseases.

It has been suggested that apoptotic cells can release a specific death secretome as a protective “farewell signal” to the environment [11, 12], and this may also apply to transplanted NSPCs. In fact, enhanced neuroprotection has been observed even in the absence of neuronal differentiation [13, 14]. It was shown, for example, that NSPCs are able to promote endogenous neural stem cell proliferation and angiogenesis, thereby indirectly contributing to functional recovery [15–17]. Further, undifferentiated NSPCs can repress chronic inflammation in the central nervous system (CNS) by inducing apoptosis of blood-borne CNS-infiltrating T cells, ultimately promoting CNS restoration [18–20]. Therefore, the secretome of dying NSPCs may contain components that are actively released and exert neuroprotective bystander effects. Here, we report that conditioned medium (CM) obtained from apoptotic NSPCs mediates protective effects to neurons at risk in both in vitro and in vivo model systems relevant to acute neurological diseases.

## Materials and methods

### Chemicals and reagents

All standard chemicals were obtained from Sigma-Aldrich (Taufkirchen, Germany) and Carl Roth (Karlsruhe, Germany). All buffers and solutions were prepared using demineralized, ultrapure water and were sterilized using a 0.22-μm filter set (Sarstedt, Nümbrecht, Germany). Recombinant human peroxiredoxin-1 (prdx-1, Sigma-Aldrich) and recombinant mouse galectin-1 (R&D Systems, Wiesbaden-Nordenstadt, Germany) were dissolved in Earle’s Balanced Salt Solution (EBSS) and used at final concentrations of 12.5–125 µg/ml and 10 µg/ml, respectively. The cell-permeable, irreversible, pan caspase inhibitor Q-VD-OPh (Merck, Darmstadt, Germany) was dissolved in dimethyl sulfoxide (DMSO) and used at a final concentration of 20–40 µM.

### Animals and surgical procedures

Seven-day-old male C57BL/6 mice (with their dams) were purchased from Charles River Laboratories (Sulzfeld, Germany). Unilateral hypoxia-ischemia (HI) was induced on postnatal day 9 (PND9) using the Vannucci model, as described previously [21]. In brief, the mice were anesthetized with isoflurane (5% for induction and 1.5% for maintenance). The left common carotid artery was isolated and ligated with prolene sutures (6–0). After a 1-h recovery period, the mice were placed in a chamber perfused with a humidified gas mixture (10% oxygen in nitrogen) at 36 °C for 50 min. All experiments were approved by the Ethics Committee of Karolinska Institutet (application number N249/13). All the purchased healthy pups were included in the study. The exclusion criteria were bleeding during the operation or death during hypoxia or transplantation surgery. After HI insult, the pups in each litter received an arbitrary number, and were sequentially allocated to different groups based on the numbers they were assigned.

### Neural stem and progenitor cell (NSPC) transplantation

NSPCs were kindly provided by Fred H. Gage (Salk Institute, La Jolla, CA) [22] and were cultured at a density of 3 × 10^5^ cells per 25 mm^2^ in DMEM/F12 (PAA, Cölbe, Germany) in a standard humidified incubator at 37 °C with 5% CO_2_ and expanded as a monolayer in uncoated tissue culture flasks (Sarstedt, Nümbrecht, Germany) as previously described [8]. Cells were routinely tested for mycoplasma infection. To track viable cells, CM-DiI was added to the culture medium at a concentration of 2 µM just before transplantation in accordance with the manufacturer’s instructions (Cat#: C7001, Invitrogen Corporation, San Diego, CA). A total of 2 × 10^5^ NSPCs in 5 µl Dulbecco’s modified Eagle’s medium (DMEM) without growth factors was injected into either the lateral vehicle or hippocampus1 h before HI was induced in the ipsilateral hemisphere. animals were sacrificed 6 h (*n* = 5 in both lateral vehicle group and hippocampus group), 24 h (*n* = 5 in both lateral vehicle group and hippocampus group) or 7 days (*n* = 12 in lateral vehicle group and *n* = 9 in hippocampus group) after HI to evaluate the viability of transplanted cells as well as the effects on brain damage. In contrast, vehicle-treated animals only received 5 µl EBSS in the lateral vehicle (*n* = 10) or hippocampus (*n* = 8) and were sacrificed 7 days after HI.

### Immunohistochemistry

Animals were deeply anesthetized with an overdose of sodium pentobarbital (50 mg/kg Pentothal, Electra-box Pharma, Stockholm, Sweden) and transcardially perfused with ice-cold PBS followed by 4% paraformaldehyde. The brains were removed, embedded in paraffin, and 5-µm serial coronal sections were cut and mounted on glass slides. After deparaffinization and rehydration, the sections were boiled in 10 mM sodium citrate buffer (pH 6.0) for 10 min to unmask epitopes. To prevent nonspecific binding, the sections were incubated in blocking solution (PBS containing 3% donkey serum and 0.1% Triton X-100) for 1 h at room temperature (RT). After blocking, the sections were incubated overnight at 4 °C with primary antibodies (mouse anti-MAP2, 1:1000, Sigma, Cat#: M4403; rabbit anti‒active caspase-3, 1:250, Cell Signaling Technology, Cat#: ASP175) diluted in blocking solution. The following day, the sections were rinsed three times with PBS, then incubated with biotinylated donkey anti-mouse IgG (1:250, Jackson ImmunoResearch, West Grove, PA, Cat#: 715-065-151) or donkey anti-rabbit (1:250, Jackson ImmunoResearch, Cat#: 711-005-152) for 1 h at RT. Endogenous peroxidase activity was blocked with 3% H_2_O_2_ in PBS for 30 min. The signal was visualized using Vector ABC Elite (Vector Laboratories, Burlingame, CA, USA, Cat#: PK-6100) and 3,3'-diaminobenzidine (DAB) enhanced with 15 mg/ml ammonium nickel sulfate, 2 mg/ml beta-D-glucose, 0.4 mg/ml ammonium chloride, and 0.01 mg/ml beta-glucose oxidase (all from Sigma).

### Cell counting and brain damage evaluation

The number of CM-DiI labeled cells was counted at 200X magnification in every 50th section, with 4 sections per animal (LSM 700; Zeiss, Oberkochen, Germany). The total number of cells was calculated by multiplying the number of positive cells per section by the number of sections sampled. The volume of MAP2-positive tissue was measured in both hemispheres. The volume of tissue loss was defined as the MAP2-positive volume in the contralateral hemisphere minus the ipsilateral hemisphere and was calculated according to the Cavalieri principle using the following formula: *V* = Σ*A* * *P* * *T*, where *V* is total volume, Σ*A* is the sum of the areas measured, *P* is the inverse of the sampling fraction, and *T* is section thickness. Infarct volume was identified as the MAP2-negative volume in the ipsilateral hemisphere. The counting of CM-DiI positive cells and measurement of brain injury were performed by a person who was blinded to the grouping.

### Cell cultures and CM preparation

Human mesencephalic stem cells (Mesc), SNL 76/7 feeder cells (SNL), human dopaminergic neurons (Dopam), human neural progenitor cell line (VM), neural stem cell line (C17.2) and HT22 mouse hippocampal neuronal cell line were cultured in a standard humidified incubator at 37 °C and 5% CO2 in 75 cm^2^ or 175 cm^2^ culture flasks and passaged when the cultures reached around 70% confluence. Primary mouse embryonic fibroblasts (MEFs) were obtained from C57BL/6 mouse embryos (embryonic day 17–18) in accordance with established protocols [23]. In brief, small pieces of embryonic skin were dissected and placed in MEF culture media. When the fibroblasts began to expand out of the pieces of skin, the cells were dissociated using a standard trypsin/EDTA solution and seeded in a new culture flask to establish a well-distributed monolayer of cells.

For harvesting of CM, cells were grown in 75 cm^2^ or 175 cm^2^ culture flasks until they reached ~70% confluence. To induce starvation, the cells were cultured in EBSS deprived of growth factors (Sigma-Aldrich) for certain time periods. The duration was cell type-dependent: NSPCs were treated for at least 24 h, and the other cell types were treated for approximately one week. The CM were then collected and stored at −80 °C until further use. Prior to the experiments, CM was centrifuged at 1000 rpm for 10 min, and the supernatant was filtered through a 0.22-μm membrane filter to remove dead cells and cell debris and heated at temperatures up to 60 °C for 10 min. To determine the protective effects, CM was applied in HT22 cells for 6 h (unless otherwise stated), followed by glutamate stimulation in the continued presence of CM.

For acetone precipitation, CM was mixed with 4 volumes of cold acetone and stored at −20 °C overnight. The following day, the solution was centrifuged at 13,000 *g* at 4 °C for 30 min. The supernatant was removed, and the pellet was resuspended in EBSS. The acetone was removed from the pellet and the supernatant by evaporation at 40 °C. A 10-kDa or 50-kDa cut-off filter was applied for size-exclusion filtration (Millipore, Schwalbach, Germany). The flow through was concentrated by centrifugation at 4000 rpm (4 °C) and stored at −80 °C until further use. To prepare cell lysates, NSPCs and SNL cells were lysed on ice in EBSS with a Digital Sonifier (VWR, Darmstadt, Germany) for 2 min at 20% amplitude (with 1-s on/1-s off cycles).

### Cell viability assays

Quantification of cell viability in HT-22 cells was performed in 96-well plates by MTT (Sigma–Aldrich, Munich, Germany) reduction at 0.25 mg/ml for 1 h. The reaction was terminated by removing the media and freezing the plate at −80 °C for at least 1 h. After solving the MTT dye in dimethyl sulfoxide (DMSO) the absorbance was determined at 570 nm, background was determined at 630 nm and subtracted accordingly (FluoStar, BMG Labtech, Offenburg, Germany). Additionally, cell death was detected through Annexin V/propidium iodide (PI) staining using an Annexin-V-FITC Detection Kit followed by FACS analysis. Annexin-V-FITC was excited at 488 nm and emission was detected through a 530 ± 40 nm band pass filter (Green fluorescence). Propidium iodide was excited at 488 nm and fluorescence emission was detected using a 680 ± 30 nm band pass filter (red fluorescence). Data were collected from 10,000 cells from at least four wells per condition.

Further, impedance-based real time detection of cellular viability was conducted using the xCELLigence system (Roche Diagnostics, Penzberg, Germany) as previously described [24]. Briefly, ~8000 HT22 cells or 30,000–40,000 NSPCs were seeded per well into 96-well E-plates. The impedance readout as recorded by the xCELLigence system is expressed as arbitrary cell index-values. The normalization of cell index (NCIti) arbitrarily sets the cell index values to 1 at the indicated time points. All following normalized values are calculated using the equation: NCIti = CIti/CInml_time. Accordingly, the NCIti is calculated as the cell index CIti at a given time point divided by the cell index at the normalization time point (CInml_time). Background impedance caused by the media was determined in each well before seeding the cells and subtracted automatically by the RTCA software following the equation: CI = (Zi − Z0)/15 with Zi as the impedance at any given time point and Z0 as the background signal. Treatment of cells was started when the cell index exceeded a value of 1. Cell index values were recorded using RTCA Software 1.2 (Roche Diagnostics, Penzberg, Germany).

All in vitro experiments were at least repeated three times and the independent replicates per treatment group varied between 3–4 (FACS analysis) and 6–8 replicates for MTT assays and xCelligence assays.

### CM injection and brain damage evaluation

Three different volumes of CM, 5 µl (*n* = 6), 10 µl (*n* = 5), or 20 µl (*n* = 23), was administered by intracerebroventricular (i.c.v.) injection at a rate of 1 µl/min just prior to HI insult. EBSS-treated animals received identical amounts of EBSS (*n* = 5 in 5 µl EBSS group, *n* = 5 in 10 µl EBSS group and *n* = 17 in 20 µl EBSS group) prior to HI insult. The coordinates for injection into the lateral ventricle of PND9 mice were as follows: 0.2 mm posterior and 1.0 mm left to bregma, at a depth of 2.5 mm below the dura. Animals were sacrificed at 24-hour time point. Animals subjected to the HI procedure only and sacrificed at PND 10 were used as controls (*n* = 6). The number of active caspase-3‒positive cells was counted in the ipsilateral cortex and hippocampus by a person who was blinded to the grouping. Cells were counted at 200X magnification in every 50th section, with 4 sections per animal (Stereo Investigator; MicroBrightField, Colchester, VT, USA). The total number of cells was calculated by multiplying the number of positive cells per section by the number of sections sampled. Results are reported as the number of positive cells in the total volume measured.

### Digestion of RNA and proteins in CM

RNA was digested with ribonuclease A (RNase A) solution (Sigma-Aldrich) at a concentration of 70k unit/mg protein. Then, RNase A activity was inactivated by heating CM at 60 °C for 30 min before further applications in cell culture. Proteases (Qiagen, Hilden, Germany) were used to digest the proteins in the CM. The lyophilized protease powder was first dissolved in EBSS, and 45 mAU/mg protein was added to the CM. The CM and protease mixture was incubated for 30 min at 37 °C, after which the proteases were inactivated by incubation at 75 °C for 4 h.

### Western blot analysis

The protein concentration was determined using the Pierce BCA kit (Perbio Science, Bonn, Germany). Samples were mixed with an equal volume of concentrated (3×) sodium dodecyl sulphate polyacrylamide gel electrophoresis (SDS–PAGE) buffer and heated (96 °C) for 5 min. Pooled samples were run on 4–20% Tris–glycine gels (Novex, San Diego, CA, USA) and transferred to reinforced nitrocellulose membranes (Schleicher & Schuell, Dassel, Germany). After blocking with 30 mM Tris–HCl (pH 7.5), 100 mM NaCl and 0.1% Tween 20 (TBS-T) containing 5% fat-free milk powder for 1 h at 25 °C, the membranes were incubated with primary antibodies: anti-caspase 3, anti-cleaved caspase 3, anti-PARP (1: 1000; Cell Signaling Technology, Danvers, USA). After washing, the membranes were incubated with appropriate secondary antibodies (Vector Laboratories, Burlingame, USA) for 30 min at 25 °C. Immunoreactive species were visualized using the Super Signal West Dura substrate (Pierce, Rockford, IL, USA) and a LAS 3000 cooled CCD camera (Fujifilm, Tokyo, Japan).

### MALDI-TOF analysis

The samples were concentrated using Amicon Ultra 10,000 MWCO filtration (Millipore, Danvers, MA) at 4300 U/min and digested with sequencing-grade modified trypsin (Promega, Madison, WI). The resulting peptide mixtures were analyzed by nanoLC (PepMap100 C-18 RP nanocolumn and UltiMate 3000 liquid chromatography system, Dionex, Thermo Scientific, Sunnyvale, CA) and automated MS/MS (4800 Proteomics Analyzer MDS, Sciex, Ontario, Canada). MS/MS data were screened against an in-house *Mus musculus* protein database using Mascot embedded in GPS explorer software (MDS Sciex). For interpretation, focus was set on the peptides with a total ion score >45 and a minimum of 2 peptide counts. The ion score for an MS/MS match is based on the calculated probability that the observed match between the experimental data and the database sequence is a random event. The reported score is −10Log(P). Thus, during a search, if 1500 peptides fell within the mass tolerance window about the precursor mass, and if the significance threshold is set to 0.05, this translates to a score threshold of 45. For interpretation, fragmentation patterns were determined by performing a BLAST search in the PubMed database.

### Statistical analysis

Our experimental design on animal size was based on previous published studies, instead of using statistical methods [25, 26]. The normality of the data was confirmed by the D’Agostino and Pearson normality test using Prism software. For the data with normal distribution and homogeneity of variance, statistical comparisons between two groups were performed using Student’s *t*-test. If normal distribution was not detected, the Mann-Whitney *U*-test was used to compare two groups. For in vitro experiments evaluating cell viability sample sizes were chosen based on previously published work, and each treatment group consisted of 8 biological replicates. Each experiment was independently repeated at least 3 times for validation of the results [27, 28]. Differences between treatment groups in vitro were analyzed using an analysis of variance (ANOVA) followed by Scheffé’s post hoc test. A *p*-value of <0.05 was considered to indicate a significant difference between compared mean values. Data are presented as the mean ± standard deviation (SD).

## Results

### Apoptotic NSPCs exhibit neuroprotective effects

**Fig. 1:**
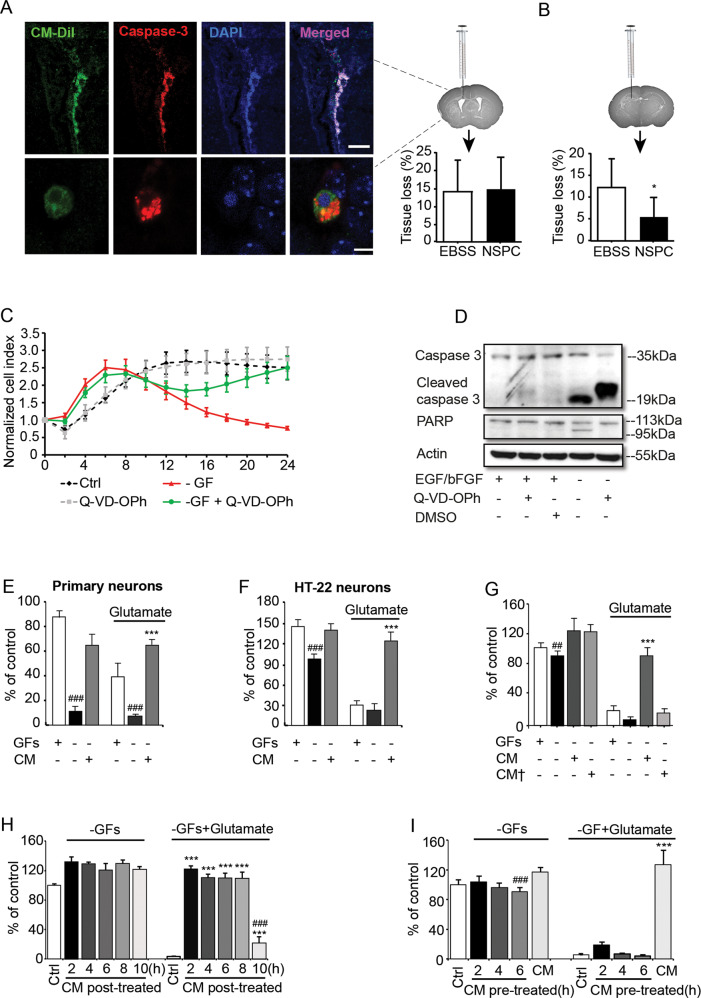
Apoptotic NSPCs exert neuroprotective effects. **A** Left panel: i.c.v. transplanted NSPCs at 6 h after HI, high-magnification images represent an apoptotic NSPC; Right panel: Brain tissue loss was not altered after ventricular NSPC transplantation as evaluated at 7 days after HI. **B** Intrahippocampal NSPC transplantation significantly reduced HI-induced brain tissue loss (%) at 7-day time point, *p < 0.05 versus the EBSS-treated group. **C** NSPC viability as determined by cellular impedance dropped distinctively over 24 h after GFs deprivation, which was reversed by Q-VD-OPh. **D** Representative western blot image indicated elevated caspase-3 and PARP cleavage in GF-deprived NSPCs, with PARP cleavage got completely blocked in the presence of Q-VD-OPh. **E**–**I** Cell viability quantified by MTT assay: CM protected primary neurons (**E**) and HT22 cells (**F**) from both GFs withdrawal and glutamate toxicity, ^###^*p* < 0.001 versus corresponding control groups; ***p < 0.001 versus the –GFs + glutamate group. **G** Conditioned medium of apoptotic NSPCs rescued by Q-VD-OPh (CM†) provided no protective effect against glutamate toxicity, ^##^*p* < 0.01 versus the control grou*p;* ***p < 0.001 versus the –GFs + glutamate group. **H** CM was added to HT22 cells that subsequently underwent glutamate stimulation, cell viability was measured at the indicated time points. NSPC-derived CM protected HT22 cells from glutamate-induced excitotoxicity for up to 8 h. ***p < 0.001 versus the –GFs + glutamate group; ^###^*p* < 0.001 versus the 2, 4, 6, and 8-hour post-treatment groups. **I** Cells were pretreated with CM for the indicated time periods, after which the CM was washed out and the cells were stimulated with 3 mM glutamate for 14 h. CM co-treatment was used as a positive control, ^###^*p* < 0.001 versus the control group; ***p < 0.001 versus the –GF + glutamate group.

A total of 2 × 10^5^ growth factor (GF)-deprived NSPCs were injected into the lateral ventricle of postnatal day 9 mice 1 h before hypoxia-ischemia (HI). The majority of these cells were positive for active caspase-3 already 6 h after injection, indicating that they were undergoing apoptosis (Fig. 1A). Fewer than 2% of the transplanted cells could be detected in the brain after 7 days (not shown). In addition, brain tissue loss after HI was not altered in transplanted animals compared to vehicle controls (15.0 ± 8.7% *vs* 13.9 ± 9.3%; *p* = 0.77. Fig. 1A). To maximize cell delivery to the injured tissue, we next injected NSPCs into the ipsilateral hippocampus, the region most susceptible to ischemic injury, immediately before HI and examined brain injury after 7 days. Again, very few cells survived the transplantation procedure (2.1 ± 1.1%), but brain tissue loss after HI was reduced by more than half in transplanted animals compared to vehicle controls (5.7 ± 5.2% *vs* 12.2 ± 6.6%; *p* < 0.05. Fig. 1B). Then, we mimicked the in vivo NSPC conditions by removing GFs from cultured NSPCs as previously described [24], and cell death was observed within 24 h (Fig. 1C). This cell death was associated with PARP cleavage by activated caspase-3, and prevented by adding the pan-caspase inhibitor Q-VD-OPh (Fig. 1C, D). To examine whether the dying NSPCs released protective factors, conditioned medium (CM) was collected and applied to primary neurons and HT22 cells (a hippocampal neuronal cell line). Both cell types were protected from trophic factor withdrawal and glutamate-induced toxicity (Fig. 1E, F). Strikingly, medium obtained from Q-VD-OPh-rescued NSPCs failed to exert any protection from glutamate toxicity (Fig. 1G). Since Q-VD-OPh per se had no effect on the viability of HT22 cells under conditions of oxidative damage, we conclude that caspase-dependent NSPC death is essential for the accumulation of protective factors in the CM. Notably, NSPC exposed to QVD or rescued from apoptosis induced by trophic factor withdrawal kept their stemness character as judged by nestin staining (Fig. S1). CM fully protected HT22 cells from cell death even when applied up to 8 h after onset of glutamate stimulation (Fig. 1H). In contrast, CM-pretreated HT22 neurons, followed by a washout period, were again susceptible to glutamate toxicity, suggesting that specific components in CM must be present for the sustained protective effects (Fig. 1I).

### Heat-resistant proteins mediate the protective effects of CM from apoptotic NSPCs

**Fig. 2:**
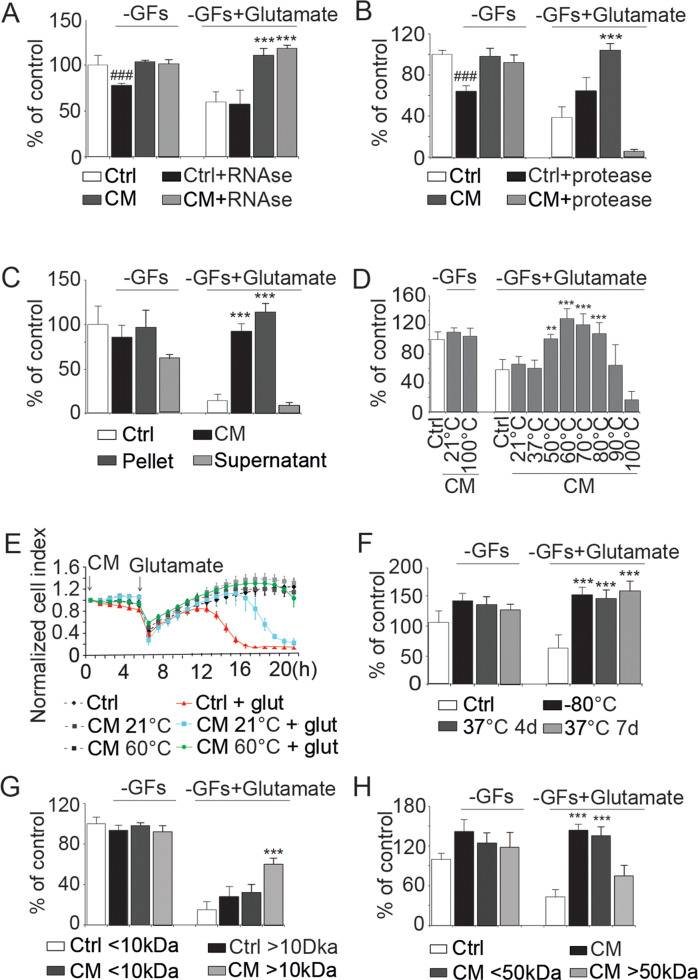
Heat-stable proteins mediate neuroprotective effects of conditioned medium from dying NSPC. Neuroprotective effects of CM were measured by MTT (**A**–**D**, **F**–**H**) and impedance assays (**E**). **A** CM treated with RNase did not affect its ability to protect HT22 cells from glutamate-induced toxicity (measured 15 h after glutamate stimulation), ***p < 0.001 versus glutamate-treatment. **B** Proteases fully prevented the protective effect of CM against glutamate-induced toxicity (measured 18 h after glutamate stimulation), ^###^*p* < 0.001 versus the control group; ***p < 0.001 versus the –GF + glutamate group. **C** Acetone precipitated CM (pellet) was dissolved in EBSS and was tested, together with supernatant, for their ability to protect HT22 cells from glutamate-induced toxicity (measured 16 h after glutamate stimulation); EBSS and CM were used as negative and positive controls, respectively, ***p < 0.001 versus the –GF + glutamate group. **D** CM was diluted (1:4), incubated at the indicated temperatures for 30 min, and tested for the ability to protect HT22 cells from glutamate-induced toxicity (*n* = 6). **p < 0.01 and ***p < 0.001 versus the –GF + glutamate group. **E** Incubating CM at 60 °C for 10 min provided significantly longer-lasting protection from glutamate-induced toxicity compared to CM that was incubated at room temperature (RT). **F** CM was heated at 60 °C for 10 min followed by incubation at 37 °C for 4 or 7 days, then tested for its ability to protect HT22 cells from glutamate-induced toxicity (measured 19 h after glutamate stimulation); NSPC-derived CM stored at -80 °C was used as a positive control. ***p < 0.001 versus the –GF + glutamate group. **G**, **H** Filtrates and concentrates of CM obtained using either 10-kDa (**G**) or 50-kDa (**H**) cut-off filter were tested for their ability to protect HT22 cells from glutamate-induced toxicity. The fractions were heated at 60 °C for 10 min before being applied to HT22 cells. ***p < 0.001 versus the –GF + glutamate group.

CM obtained from stem cells may contain various protective factors, such as metabolites, RNA and proteins, exosomes or even organelles [12, 29–31]. To identify the specific protective components, CM was treated with RNase and tested on glutamate-stimulated HT22 cells. RNase treatment did not affect the protective effects of CM (Fig. 2A). On the contrary, pretreating CM with proteases abolished its protective effects (Fig. 2B). In the CM of Q-VD-OPh-treated cells the pan-caspase inhibitor present in this medium may still exert protective effects because of caspase inhibition after GF withdrawal as also demonstrated in Fig. 1C. The protease inhibitors may also inhibit adverse signalling molecules released into the medium after GF withdrawal. However, both treatments did not rescue from glutamate toxicity and the protease incubation fully blocked the protective effect of the CM from cells dying after GF withdrawal. Given that proteases can induce nonspecific cytotoxicity, including the accumulation of toxic protein degradation products, an additional approach to remove proteins and peptides by acetone precipitation was conducted. EBSS-resuspended precipitated proteins, but not protein-free supernatant, exerted the same effect as the original CM (Fig. 2C). Treating NSPCs with the protein synthesis inhibitors actinomycin D or cycloheximide did not alter the protective properties of the resulting CM (Fig. S2), indicating that the neuroprotective proteins were not merely stress-induced, but constitutively synthesized in the NSPCs and released during cell death. Surprisingly, RNase inactivation at 60 °C significantly enhanced the protective potency of CM. To confirm increased protective effects through heating, CM was diluted to a level that was no longer protective (1:4), followed by heating at different temperatures. Indeed, protection re-emerged when CM was pre-incubated at 50–80 °C (Fig. 2D). Real-time impedance measurements confirmed that CM pre-incubated at 60 °C has longer-lasting protective effects compared to that incubated at room temperature (Fig. 2E). Moreover, CM heated at 60 °C and subsequently stored at 37 °C for 4 or 7 days could still protect cells from glutamate-induced toxicity, with no measurable loss of efficacy (Fig. 2F). Similar, despite less sustained, protective effects by the secretome of apoptotic NSPC were confirmed in human ReNcell ventral mesencephalon (VM) cells (Fig. S3) and mouse C17.2 neural stem cells (Fig. S4) but not in human mesencephalic stem cells (Mesc), mouse embryonic fibroblasts (MEFs), SNL feeder cells, or human differentiated dopaminergic neurons (Fig. S5A–D), suggesting that the observed effects are cell type specific and exist for neural progenitor/stem cells across species. In addition, we verified that MEFs also died in a caspase-dependent manner (Fig. S6), suggesting it is indeed the cell type and not the mode of regulated cell death that determined the protective nature of the secretome of the dying cells.

### Peroxiredoxin-1 and galectin-1 are two major protective components in NSPC CM

**Fig. 3:**
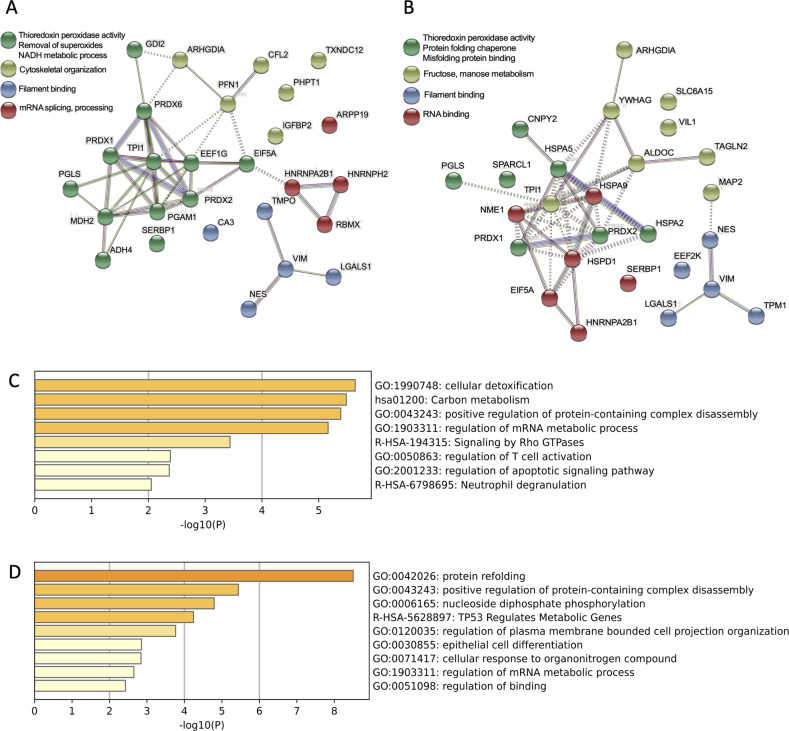
Protein pathway maps. Interaction maps of CM-containing proteins between 10 and 50 kDa visualized with STRING program (**A**) and their associated gene ontology signaling pathways, generated with Metascape (**C**). Protein interaction maps of CM-containing proteins following heating at 60 °C, generated with STRING program (**B**) and their associated pathways generated with Metascape (**D**).

Next, we performed size-exclusion filtration, incubated the fractions at 60 °C, and then tested each fraction for its ability to protect HT22 cells from glutamate-induced cell death. Only fractions containing proteins >10 kDa and <50 kDa provided protection (Fig. 2G, H). We next performed Matrix-Assisted Laser Desorption Ionization Time-of-Flight (MALDI-TOF) analyses of NSPC-derived CM, identifying 27 proteins in the molecular weight range between 10 kDa and 50 kDa that were highly expressed in CM as compared with that prepared from Q-VD-OPh-treated NSPCs. Of these, peroxiredoxins (prdxs), galectin-1 and heat shock proteins have been shown to participate in redox regulation, neuro-regeneration and stress protection, respectively (Table S1; Fig. 3). Importantly, prdx family members and galectin-1 were also detected in CM pre-incubated at 60 °C (Table S2). Analysis of other cell-specific CMs revealed that prdx-1 was present in CM samples obtained from VM cells and C17.2 cells (Tables S3, S4). In addition, galectin-1 was also identified in C17.2 cell-CM (Table S4). In contrast, only structural proteins such as vimentin and actin were identified in CM obtained from SNL feeder cells (Table S5).

**Fig. 4:**
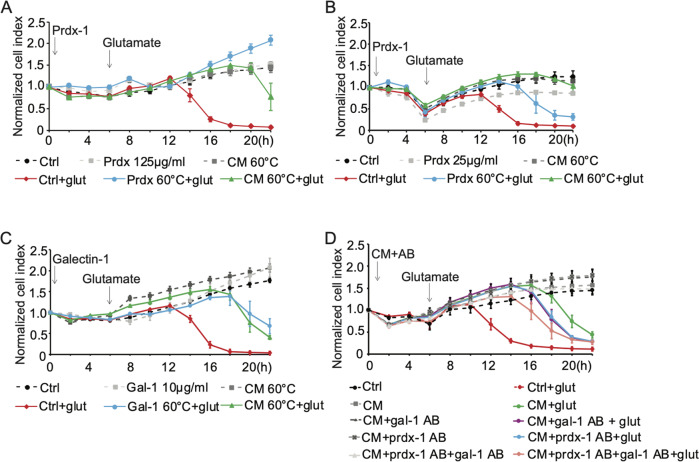
Peroxiredoxin-1 and galectin-1(gal-1) in the 10–50 kDa protein range mediate the protective effects of CM detected in real-time through impedance measurements. **A**, **B** HT22 cells were pretreated with human recombinant prdx-1 at 125 µg/ml (**A**) or 25 µg/ml (**B**). In both experiments, the recombinant protein was pre-incubated at either room temperature or 60 °C for 10 min. After 6 h, glutamate (3 mM) was added to the wells and cellular impedance was measured over time. **C** Real-time impedance measurements of HT22 cells revealed that recombinant mouse galectin-1 (10 µg/ml) provides transient protection against glutamate-induced toxicity. **D** Specific antibodies against galectin-1 and prdx-1 reduced the protective effects of CM, and combining the two antibodies exerted an additive effect.

We determined the efficacy of recombinant prdx-1 and recombinant galectin-1 in vitro and compared with that of CM. Recombinant prdx-1 prevented glutamate-induced cell death in HT22 cells in a dose-dependent manner at concentrations of 25 and 125 µg/ml (Fig. 4A, B), and recombinant galectin-1 exerted a protective effect at 10 µg/ml (Fig. 4C). The protective effects were more sustained when the proteins were pre-incubated at 60 °C. However, neither the prdx-1 levels, nor the galectin-1 levels in the CM were sufficient to fully explain the protective effect obtained by CM. For example, a reduced, albeit transient protective effect of CM remained for several hours after immunodepletion by anti-prdx-1 or anti-galectin-1 antibodies (Fig. 4D). Combining the two antibodies showed an additive inhibitory effect, but still did not abolish the protective effects of CM, suggesting the existence of additional contributory factors. Therefore, we next applied the heated conditioned medium in vivo to test the hypothesis, that the secretome of dying NSPCs is sufficient to provide protection against ischemic brain damage independent of transplantation of the NSPCs.

### CM from apoptotic NSPCs provides neuroprotective effects in vivo

**Fig. 5:**
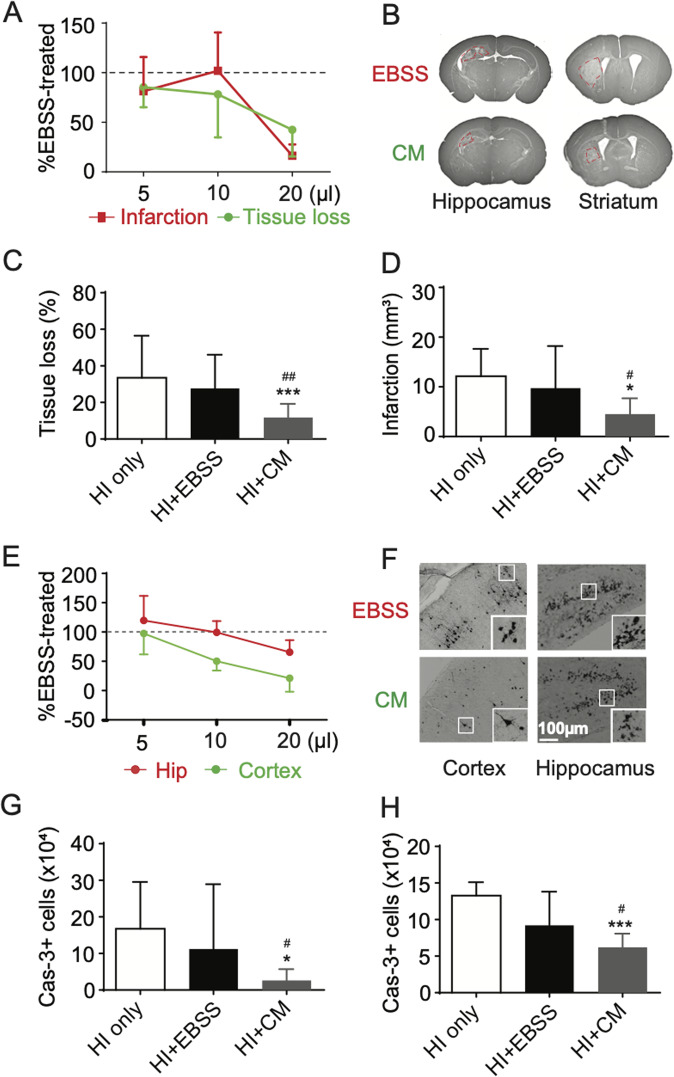
NSPC-derived CM ameliorates HI-induced brain damage. **A** The indicated volumes of NSPC-derived CM (hereafter as CM) were injected into the ipsilateral lateral ventricle prior to the induction of HI. Data are expressed as a percentage (%) relative to the corresponding vehicle-treated groups. **p < 0.01 versus EBSS-treated group in regard to tissue loss; ^#^*p* < 0.05 versus EBSS-treated group in regard to infarction volume. **B** Representative MAP2-stained coronal sections at the level of the hippocampus (left column) and striatum (right column) 24 h after HI in mice that were injected with either 20 µl vehicle (EBSS, upper row) or 20 µl CM (lower row). **C** Tissue volume loss on the ipsilateral hemisphere relative to the contralateral hemisphere, represented as a percentage (0% represents no tissue loss in the ipsilateral hemisphere). **D** Infarct volume was quantified in mice that received an intraventricular injection of 20 µl EBSS or CM prior to HI. *P < 0.05, **P < 0.01 compared with HI only group. ^#^*P* < 0.05, ^###^*P* < 0.001 compared with EBSS-treated group. *n* = 6, 17 and 23 mice in the HI only, EBSS and CM groups, respectively. **E** The number of active caspase-3-positive cells in the cerebral cortex and hippocampus (Hip) was quantified. Data are expressed as a percentage (%) relative to the corresponding vehicle-treated groups. *p < 0.05 versus EBSS-treated group in cortex; ^##^*p* < 0.01 versus EBSS-treated group in hippocampus. **F** Photomicrographs of re*p*resentative caspase-3-stained sections of cortex (left column) and dentate gyrus (right column) 24 h after HI in mice that received 20 µl of either vehicle (EBSS, upper row) or CM (lower row). The inserts show high magnification views of the boxed regions. **G**, **H** The number of active caspase-3-positive cells in the cortex (**G**), hippocampus (**H**) in mice that received an intraventricular injection of 20 µl EBSS or CM prior to HI was quantified. The data are presented as the mean ± SD. *p < 0.05, ***p < 0.001 compared with HI only group. ^#^*P* < 0.05 compared with EBSS-treated group. *n* = 6, 17 and 23 mice in the HI only, EBSS and CM groups, respectively.

Finally, we assessed the neuroprotective capacity of CM in vivo. Tissue loss, infarct volume, and caspase-3 activation were determined 24 h after HI. We found that CM provided a dose-dependent protection against HI-induced brain tissue loss. Animals that were injected with 5 µl, 10 µl, or 20 µl CM had 85.5% (17.0 ± 1.6 mm^3^ *vs* 19.9 ± 4.0 mm^3^ *p* > 0.05), 78.2% (10.3 ± 2.9 mm^3^ *vs* 13.1 ± 1.2 mm^3^ *p* > 0.05), and 55.7% (11.8 ± 1.6 mm^3^ *vs* 27.8 ± 4.1 mm^3^ *p* < 0.01) tissue loss compared with vehicle-treated littermates, respectively (Fig. 5A–C). Moreover, the infarct volumes in mice treated with 20 µl CM were significantly smaller than vehicle-treated ones (4.5 ± 0.7 mm^3^ *vs* 9.7 ± 1.9 mm^3^ *p* < 0.05) (Fig. 5A, D). CM treatment also prevented caspase-3 activation in a dose-dependent manner. Twenty microliter CM was able to decrease the number of active caspase-3‒positive neurons in both cortex (2.5 ± 0.5 × 10^4^ *vs* 11.1 ± 4.1 × 10^4^; *p* < *0.05*) and hippocampus (6.5 ± 0.4 × 10^4^ *vs* 9.8 ± 1.0 × 10^4^; *p* < *0.01*) (Fig. 5E–H). Importantly, 20 µl CM is produced by approximately 2 × 10^5^ NSPCs, which is the same number of cells injected in the NSPC transplantation experiments (Fig. 1B).

## Discussion

We identified a bystander effect of apoptotic NSPCs that mediated neuroprotection through the release of a protein secretome. Some of the protective factors from the dying cells may have been released due to late necrosis of the apoptotic cells. It is important to note, however, that caspase activity and caspase-dependent cell death was required to obtain protective CM, since both CM from cells exposed to caspase inhibition and application of cell extracts after simple cell lysis were not providing protective effects against glutamate toxicity. Further, we could not detect any substantial contribution of apoptotic bodies or cellular vesicles to the protective effect by the CM, and inhibition of cellular vesicle release did not affect full protection by the CM from the apoptotic cells. It is therefore possible, that the small proteins were either actively transported through the cell membranes as also suggested before for peroxiredoxins, or that the factors were released (in addition) after cell membrane disruption in late necrotic cells. We confirmed that the majority of transplanted NSPCs were lost after transplantation through caspase-dependent apoptosis. Thus, our findings support a new concept explaining how transplanted NSPCs protect brain tissue despite low survival rates, i.e. through a “farewell” bystander effect. Further, we demonstrate that this secretome can be harvested from apoptotic NSPCs to provide neuroprotection in vitro and in vivo independent of the presence of the dying cells. We cannot exclude that NSPC cells in vitro, after being transplanted in an inflammatory microenvironment in this model, may become apoptotic through pathways different from mere growth factor withdrawal, e.g. through activation of TNFa- or other death receptor stimulation. Therefore, the secretome from dying cells in vivo may differ from the secretome investigated from the cultured NSPC cells, However, it is important to note that the secretome from apoptotic NSPC in culture exerted protective effects, in absence of the cells, similar to cell transplantation in vivo, suggesting that the “farewell signal” from the apoptotic cells in vitro was sufficient and as effective as the “farewell signal” from dying cells in vivo, and does not require further NSPC-cell interactions.

Using MALDI-TOF, we identified a number of secreted proteins present in the secretome protein mixture. Among these, prdx-1 and galectin-1, were selected for further analysis based on their appearance in both the heated secretome fraction and the protective protein fraction >10 kDa and <50 kDa. Prdxs have peroxidase activities that reduce and detoxify hydrogen peroxide, peroxynitrite, and a wide range of organic hydroperoxides [32]. In the CNS, prdxs are believed to function as scavengers of free radicals and can protect a variety of cell types, including neurons, as reported in various models of neuronal death in vitro and in vivo [33–35]. The extracellular functions of galectins are mediated primarily through their lectin activity [36]. Galectin-1 is an important modulator in the central nervous system, regulating neuro-regeneration and neuroinflammation [37–39]. Galectin-1 in its oxidized form promotes the neurite outgrowth and enhances axonal regeneration in the picomolar range [40, 41]. By modulating the MAPK/IκB/NFκB axis through its carbohydrate-recognition domain, galectin-1 is able to inhibit microglia activation and promote neurological recovery [38]. Moreover, galectin-1-secreting neural stem cells have been shown to elicit long-term neuroprotection against ischemic brain injury [42]. In this study, neutralization of these two molecules significantly reduced the protective effects of CM, strongly implying that secretion of prdx-1 and galectin-1 by NSPCs plays a significant role in the observed protective effect. However, both factors apparently only partly contributed to neuroprotection, and other components at concentrations below the current detection limit or metabolites below a molecular size of 10 kDa may also add to the observed protective effects. For example, George et al. demonstrated that electrically preconditioned NSPCs prior to cell transplantation improved stroke recovery through the increased secretion of VEGF-A [17] and delivering VEGF-A and MMP-9 using a polymeric system enhanced stroke recovery to an equivalent degree as observed with traditional stem cell treatment [14]. More recently, a study in leukocytes demonstrated the release of an apoptotic metabolite secretome that provided cytoprotective effects, suggesting that also metabolites can mediate protective signaling from dying cells [12]. Here, we identified proteins as the major protective components released from apoptotic NSPCs, and the activity was further enhanced through heating the CM, which may have eliminated heat-sensitive chaperones counteracting the full protective effect.

NSPCs provide the opportunity to mitigate various neurological diseases through multimodal therapeutic actions. Here, we suggest a standardized protocol for harvesting and applying the protective secretome of dying NSPCs, thereby using the therapeutic potential of this concept without cell transplantation. The clinical implications of our findings are vast, enabling development of standardized apoptotic secretomes from apoptotic stem cells for treating acute brain injuries.

## Supplementary information

Original Data File

Checklist

Supplementary Figure Legends

Supplementary Figure 1

Supplementary Figure 2

Supplementary Figure 3

Supplementary Figure 4

Supplementary Figure 5

Supplementary Figure 6

Supplementary Tables
