# Correction: Dying transplanted neural stem cells mediate survival bystander effects in the injured brain

**DOI:** 10.1038/s41419-023-05730-2

**Published:** 2023-03-20

**Authors:** Wei Han, Eva-Maria Meißner, Stefanie Neunteibl, Madeline Günther, Jörg Kahnt, Amalia Dolga, Cuicui Xie, Nikolaus Plesnila, Changlian Zhu, Klas Blomgren, Carsten Culmsee

**Affiliations:** 1 Department of Women’s and Children’s Health, Karolinska Institutet, Stockholm, Sweden; 2 Henan Key Laboratory of Child Brain Injury, Institute of Neuroscience and Third Affiliated Hospital of Zhengzhou University, Zhengzhou, 450052 China; 3 Institute of Pharmacology and Clinical Pharmacy, University of Marburg, Marburg, Germany; 4 Center for Mind, Brain and Behavior (CMBB), Universities of Marburg and Giessen, Marburg, Germany; 5 Max-Planck-Institute for Terrestrial Microbiology, Department of Ecophysiology, Marburg, Germany; 6 Faculty of Science and Engineering, Molecular Pharmacology – Groningen Research Institute of Pharmacy, Groningen, The Netherlands; 7 Center for Brain Repair and Rehabilitation, Institute of Neuroscience and Physiology, University of Gothenburg, Gothenburg, Sweden; 8 Institute for Stroke and Dementia Research (ISD), University Clinic Munich, Munich, Germany; 9 Pediatric Oncology, Karolinska University Hospital, Stockholm, Sweden

**Keywords:** Cellular neuroscience, Neural stem cells

Correction to: *Cell Death and Disease* 10.1038/s41419-023-05698-z, published online 01 March 2023

The original version of this article contained an error in the author affiliations. Klas Blomgren is not affiliated with the University of Gothenburg anymore. The original article has been corrected.

