# Supplementary material for: Dying transplanted neural stem cells mediate survival bystander effects in the injured brain
Source: Cell Death Dis. 2023 Mar 1;14(3):173. doi: 10.1038/s41419-023-05698-z (PMC9975220; doi:10.1038/s41419-023-05698-z)
Supplement: Supplementary file 1 — Original Data File [file 41419_2023_5698_MOESM1_ESM.docx]

**Original Data File**

**Original scans of Western Blots**

**Figure 1**


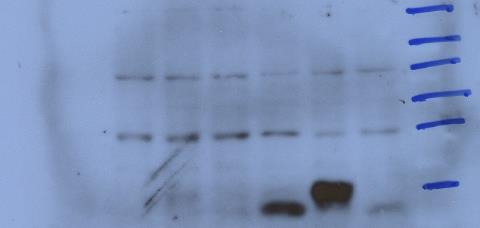


***Caspase 3***

***PARP***

***Actin***

***Cleaved caspase 3***


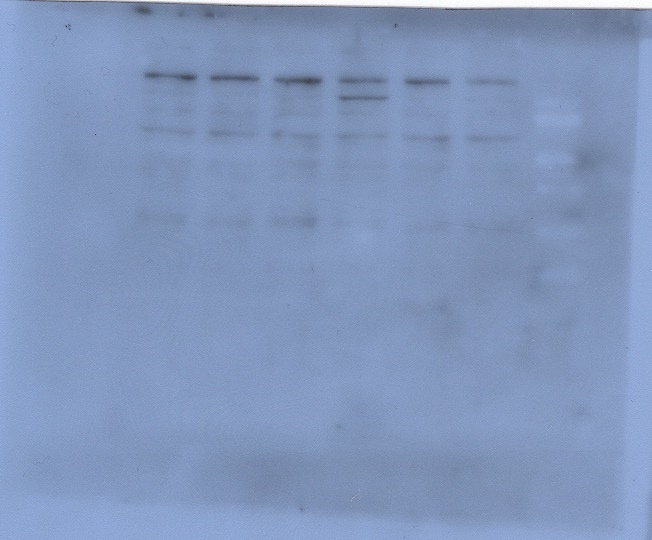

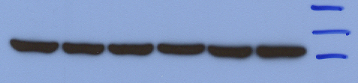


**Supplement Figure S6**

**Figure S6C**


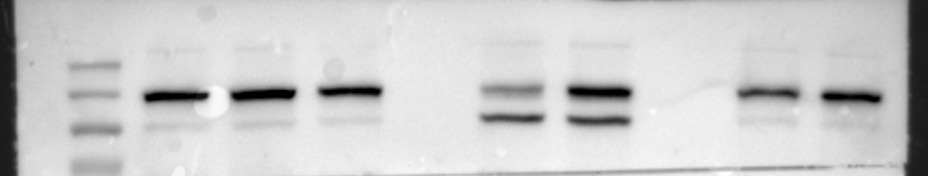


***PARP***


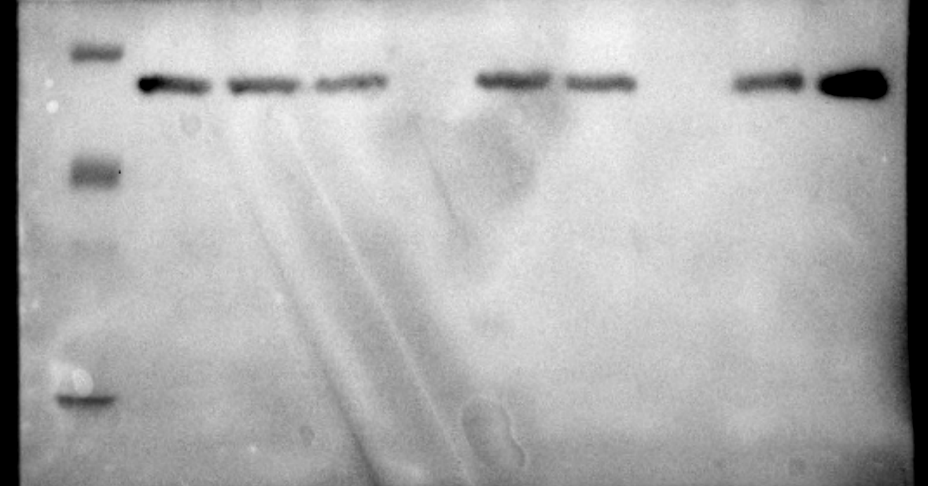


***Actin***

**Figure S6G**


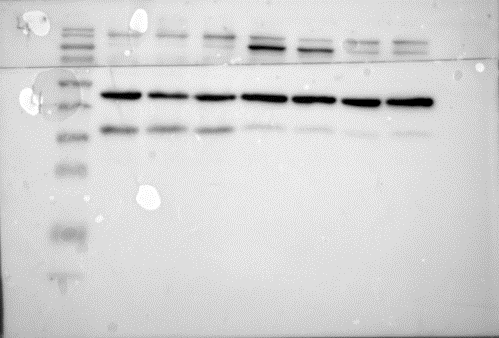


***Actin***

***PARP***
