# Supplementary material for: Dying transplanted neural stem cells mediate survival bystander effects in the injured brain
Source: Cell Death Dis. 2023 Mar 1;14(3):173. doi: 10.1038/s41419-023-05698-z (PMC9975220; doi:10.1038/s41419-023-05698-z)
Supplement: Supplementary file 3 — Supplementary Figure Legends [file 41419_2023_5698_MOESM3_ESM.docx]

Supplementary Figure Legends

**Fig. S1.** Protective effects of Q-VD-OPh against stem cell character loss under growth factor deprivation in neural stem and progenitor cells. NSPC were incubated either in growth medium containing EGF/bFGF or EBSS for 6 or 24 hours with and without Q-VD-OPh. Presence of stem cell character was detected by nestin localization on a confocal microscope (n=10), scale bar 25 μM.

**Fig. S2.** CM generated in the presence of protein synthesis inhibitors protects HT22 cells against glutamate-induced cell death. (**A, B**) HT22 cells treated with CM produced in the presence of 1 nM (**A**) and 10 nM (**B**) Actinomycin D (ACTD) prevent glutamate toxicity (3 mM, 15 h). (**C, D**) Cycloheximide (CHX) had slightly toxic effects on HT22 cells. CM produced in the presence of 0.5 nM (**C**) and 1 nM (**D**) CHX, however, still provided significant neuroprotective effect, ###p < 0.001 versus corresponding control groups; *** p < 0.001 versus the –GFs + glutamate group.

**Fig. S3.** Neuroprotective effect of CM derived from human neural progenitor cell (VM). **(A)** VM cells undergo cell death within hours of GF deprivation as determined by MTT assay, ###p < 0.001 versus the control group. **(B)** VM CM at RT failed to protect HT22 cells against glutamate toxicity (4 mM, 17 h). Whereas, heated VM CM at 60°C for 10min provided protective effects, *** p < 0.001 versus the –GFs + glutamate group. **(C)** Q-VD-OPh protected VM cells from GF deprivation (8 h) induced cell death. 100 x magnitude. **(D)** The protective effect of VM CM (incubated at 60°C) generated from cells rescued by Q-VD-OPh in HT-22 cells was significantly compromised as compared with that obtained from dying VM cells.

**Fig. S4.** Neuroprotective effects of CM derived from C17.2 neural stem cells. **(A)** Photomicrographs represent C17.2 cell death at 3 days after GFs withdrawal. **(B)** MTT assay indicated that cell viability was already reduced within 5 hours, ###p < 0.001 versus the control group. **(C)** C17.2 CM provided protective effects only when incubated at 60°C and C17.2 cell lysates provided protective properties against glutamate toxicity (4 mM, 18 h) irrespective of heat activation. ***p < 0.001 compared to the –GFs + glutamate group. **(D)** Real-time impedance measurements of HT22 cells revealed the protective potential of C17.2 CM in HT-22 cells was not as that of NSPC CM.

**Fig. S5.** Protective effects of CM from various cell types. CM harvested from cultured human mesencephalic stem cells (Mesc; **A**), primary mouse embryonic fibroblasts (MEFs; **B**), SNL feeder cells **(C)** and human dopaminergic neurons (Dopam.; **D**) failed to protect HT22 cells from glutamate-induced toxicity, measured 15 h after glutamate stimulation. ^###^*p* < 0.001 versus corresponding control groups; *** p < 0.001 versus the –GFs + glutamate group.

**Fig. S6.** Growth factor deprivation induced apoptosis in mouse embryonic fibroblasts. **(A, B)** FACS analysis for Annexin V/PI stained cells demonstrated cell death after 12 hours of growth factor deprivation in MEF cells, which was prevented moderately by Q-VD-OPh. **(C, D)** The representative Western blot with elevated cleaved PARP protein levels after 12 h indicated that MEF cells undergo apoptotic cell death after growth factor deprivation. **(E, F)** Cell death assessment with Annexin V/PI after 24 hours revealed increased cell survival of MEF cells under Q-VD-OPh treatment. **(G, H)** Cleaved PARP protein levels were analyzed by Western Blot after 24 h EBSS exposure without and in the presence of the caspase inhibitor Q-VD-OPh, the caspase inhibitor demonstrated strong protective effects against apoptosis. **(A-H)** ***p < 0.001 compared to EBSS control (ANOVA, Scheffé's test).
