# Supplementary figures and images for: Dying transplanted neural stem cells mediate survival bystander effects in the injured brain
Source: Cell Death Dis. 2023 Mar 1;14(3):173. doi: 10.1038/s41419-023-05698-z (PMC9975220; doi:10.1038/s41419-023-05698-z)

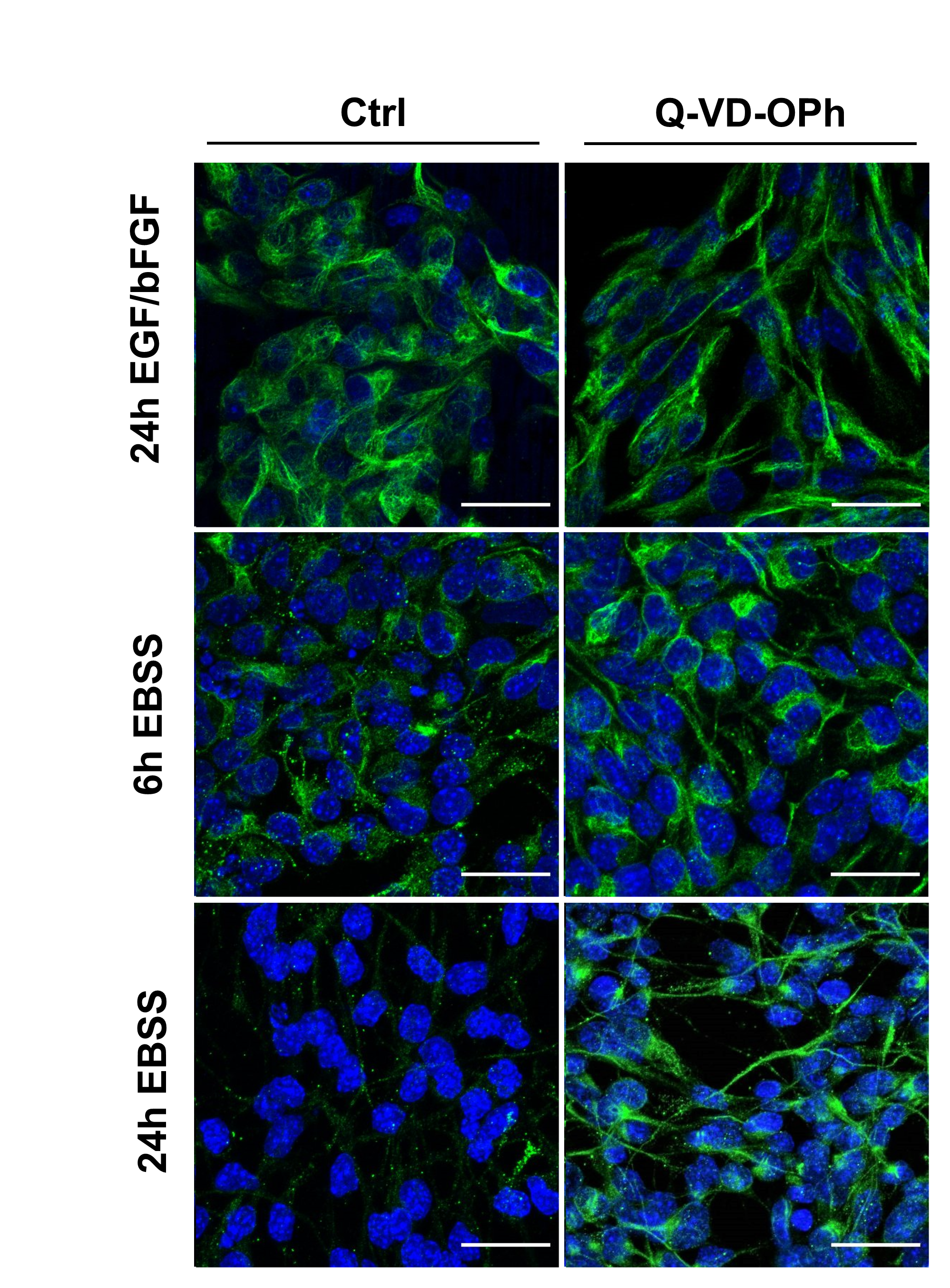

Supplement: Supplementary file 4 — Supplementary Figure 1 [file 41419_2023_5698_MOESM4_ESM.tif]

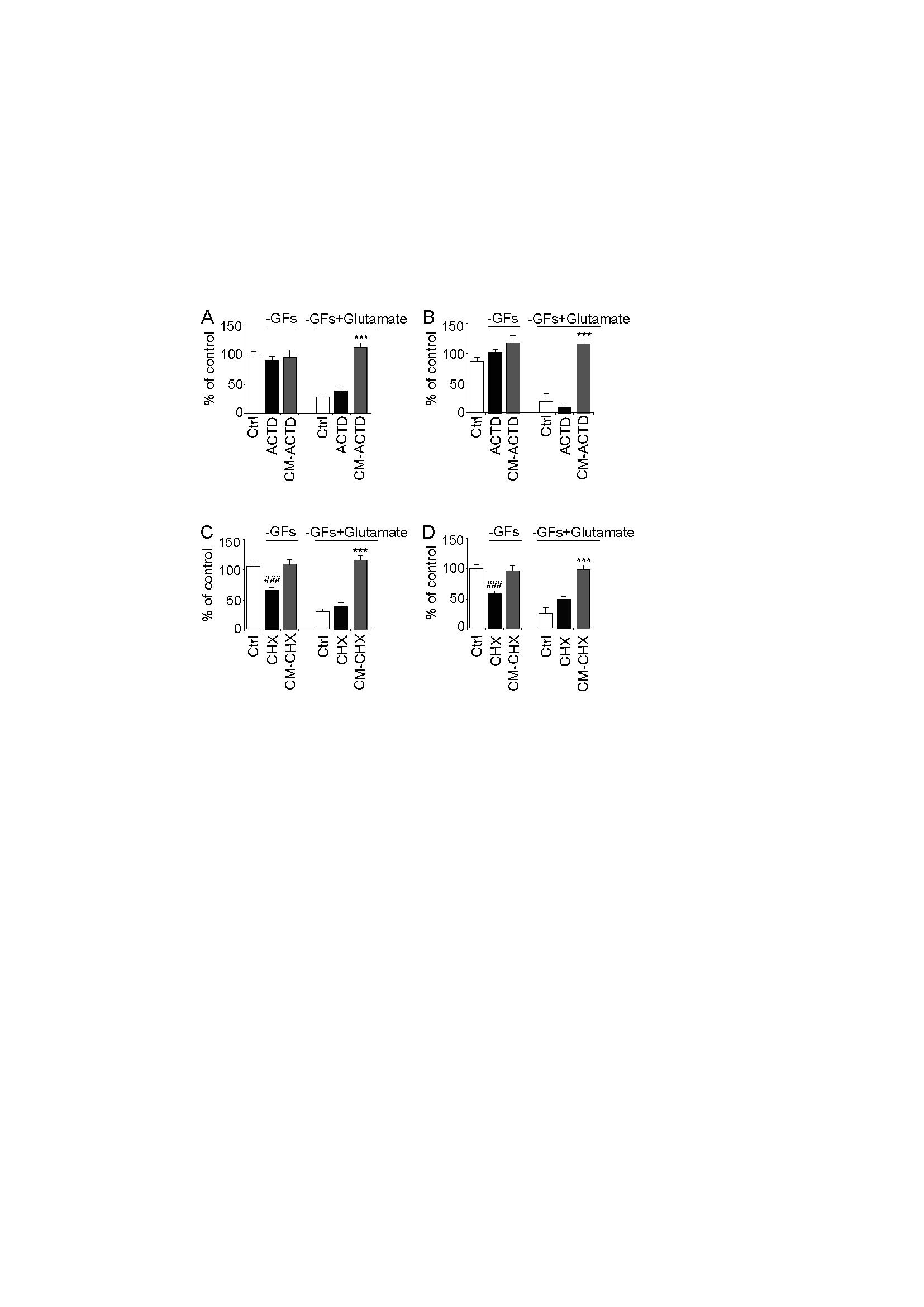

Supplement: Supplementary file 5 — Supplementary Figure 2 [file 41419_2023_5698_MOESM5_ESM.tif]

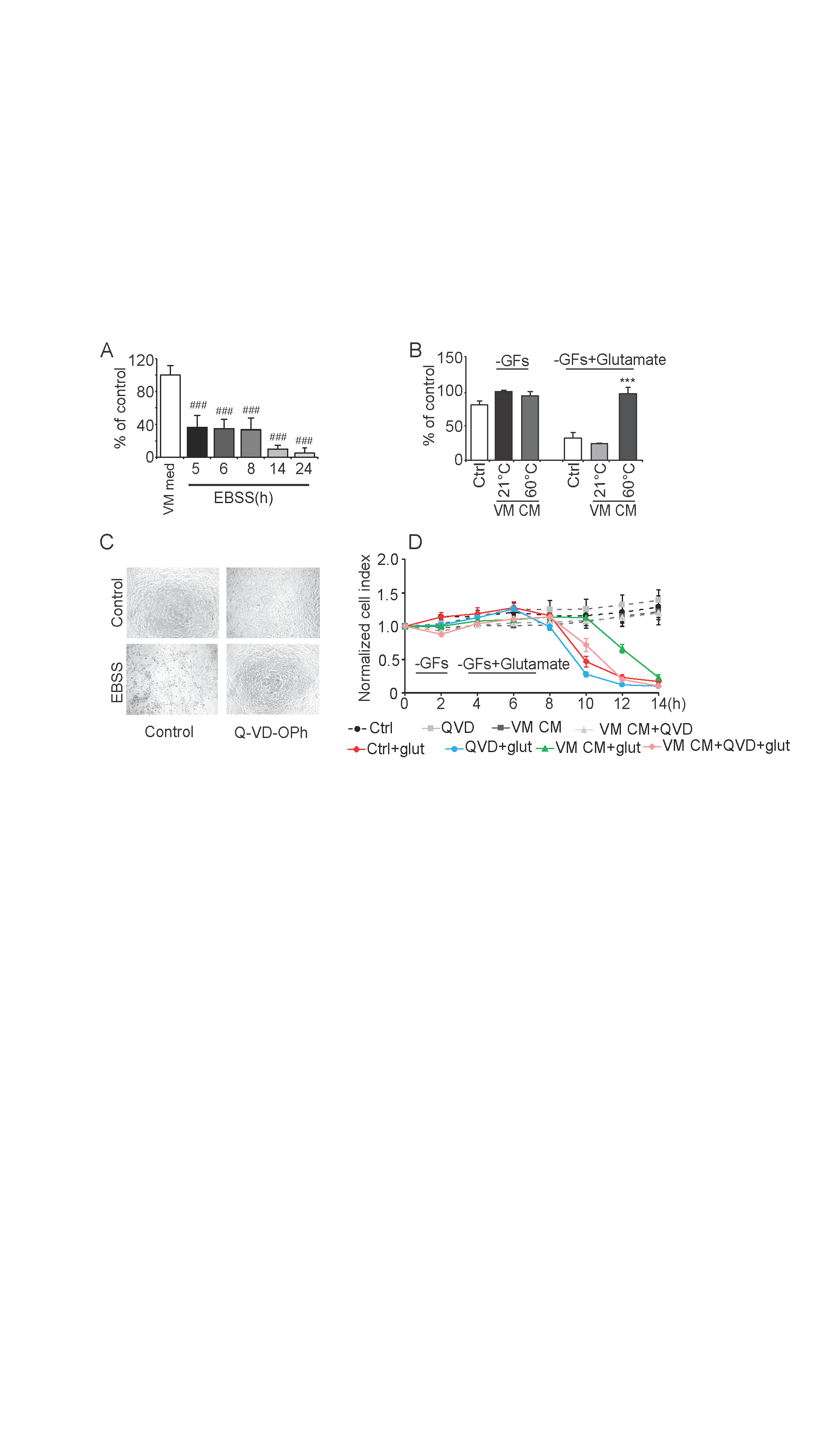

Supplement: Supplementary file 6 — Supplementary Figure 3 [file 41419_2023_5698_MOESM6_ESM.tif]

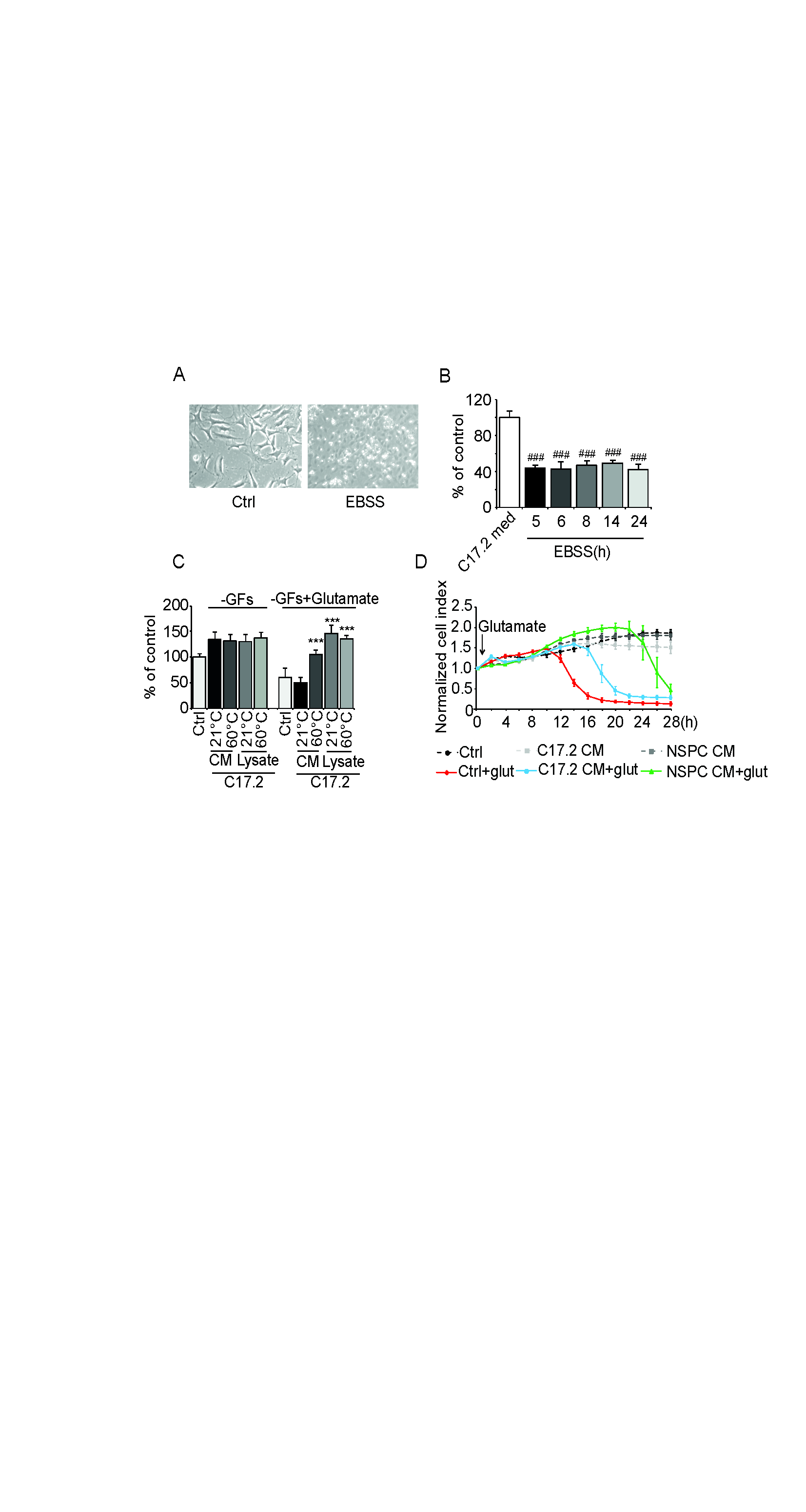

Supplement: Supplementary file 7 — Supplementary Figure 4 [file 41419_2023_5698_MOESM7_ESM.tif]

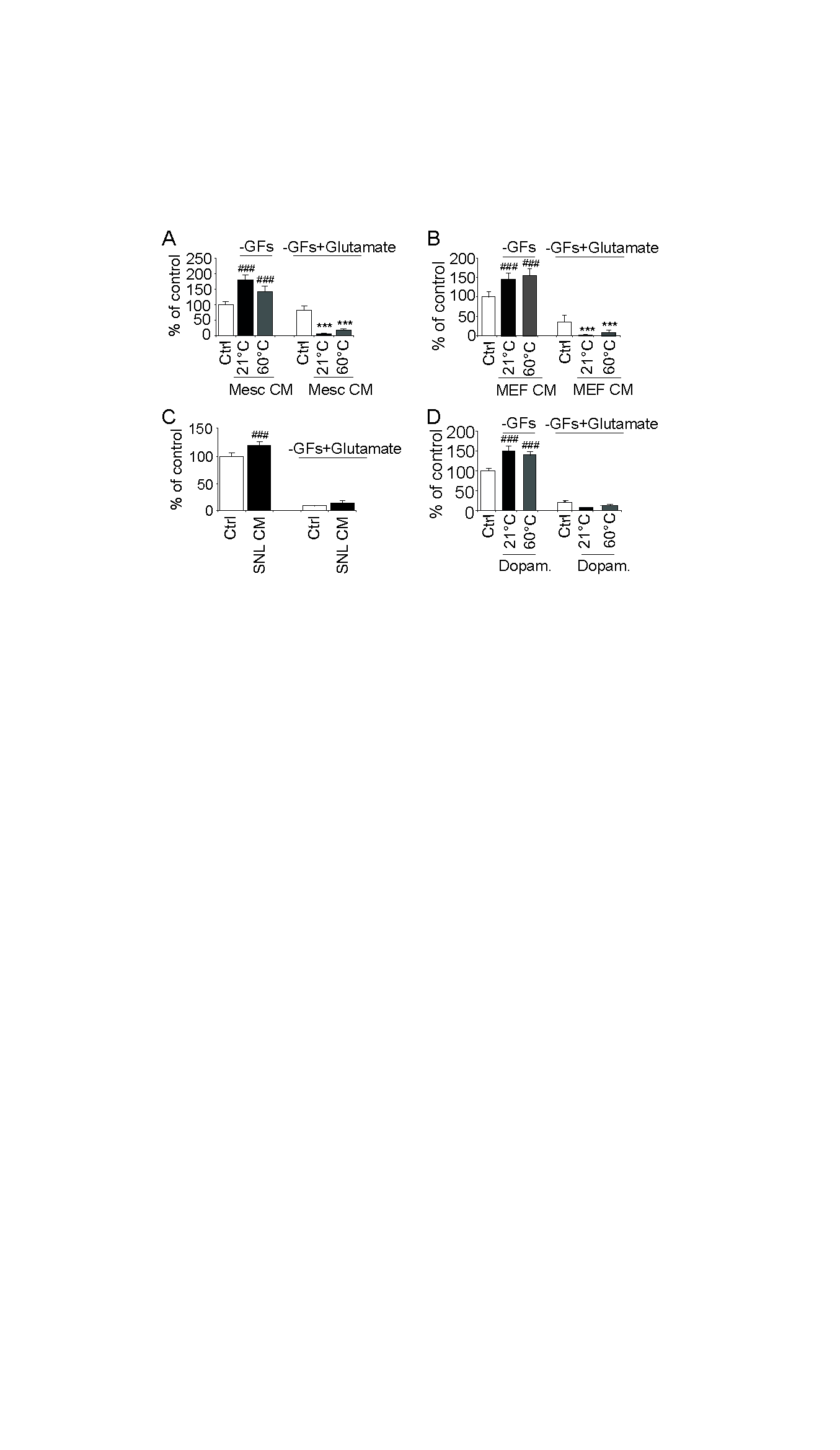

Supplement: Supplementary file 8 — Supplementary Figure 5 [file 41419_2023_5698_MOESM8_ESM.tif]

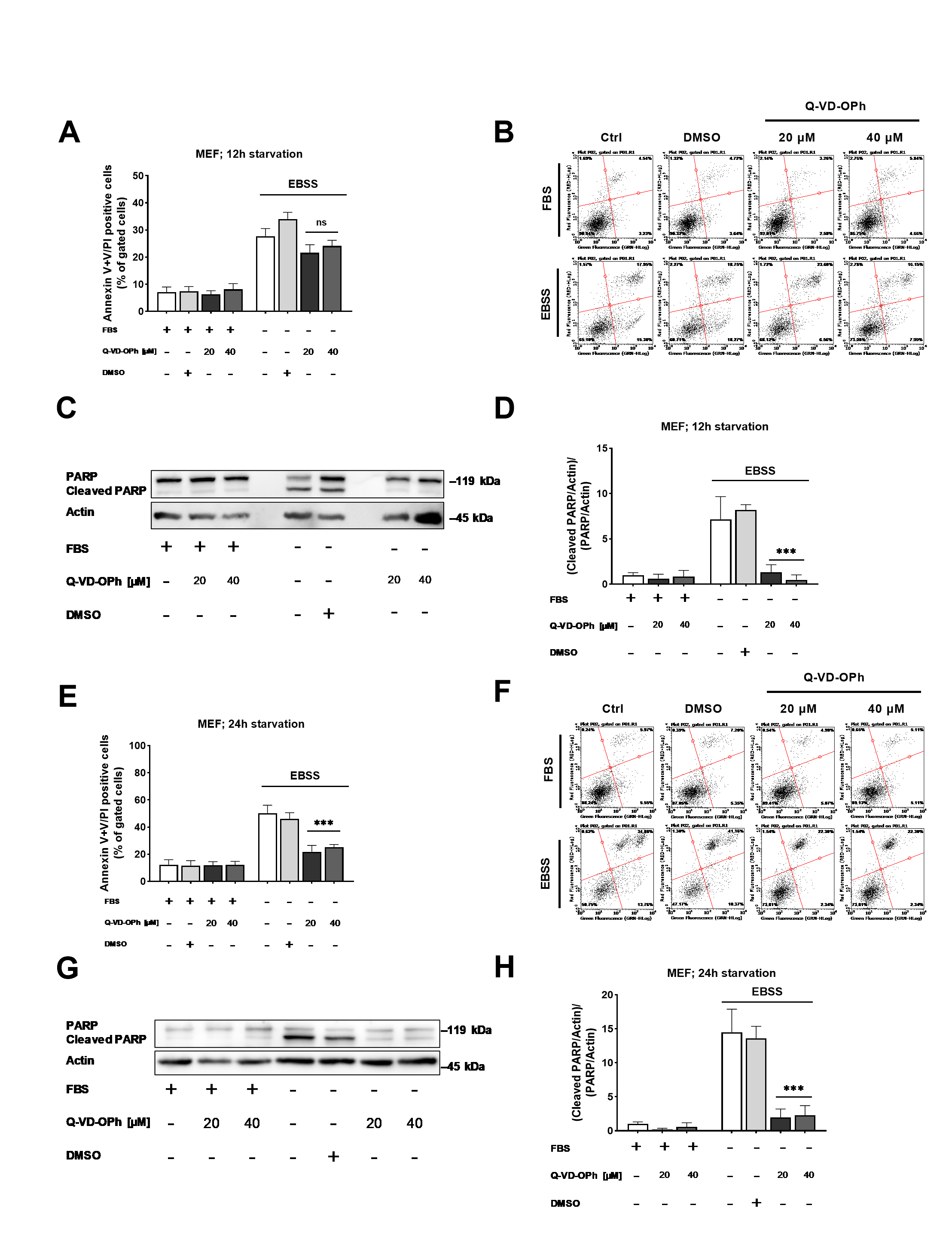

Supplement: Supplementary file 9 — Supplementary Figure 6 [file 41419_2023_5698_MOESM9_ESM.tif]
