# Supplementary material for: Dying transplanted neural stem cells mediate survival bystander effects in the injured brain
Source: Cell Death Dis. 2023 Mar 1;14(3):173. doi: 10.1038/s41419-023-05698-z (PMC9975220; doi:10.1038/s41419-023-05698-z)
Supplement: Supplementary file 10 — Supplementary Tables [file 41419_2023_5698_MOESM10_ESM.docx]

Supplementary Tables

**Table S1: MADLI-TOF analysis from CM containing proteins between 10 and 50 kDa.** Proteins with a total ion score > 45 are listed. n = number of experiments containing the named protein out of two independent experiments, two runs each.

|  | **Accession Number** | **Peptide Count** | **Total Ion Score** | **Blast** | **n** |
| --- | --- | --- | --- | --- | --- |
| 1 | P000049887\|10141..13142\|X127442\|MAUS_023456 | 11 | 893 | triosephosphate isomerase | 2 |
| 2 | P000021382\|7754..13998\|X146990\|MAUS_004980 | 9 | 702 | heterogeneous nuclear ribonucleoproteins A2/B1 isoform 2 | 2 |
| 3 | P000025352\|10111..18938\|X170045\|MAUS_004891 | 7 | 755 | nestin | 2 |
| 4 | P000021019\|10156..12512\|X144737\|MAUS_018293 | 6 | 726 | profilin-1 | 1 |
| 5 | P000052885\|10071..13417\|X174586\|MAUS_068220 | 6 | 565 | galectin-1 | 2 |
| 6 | P000026632\|10064..18153\|X177687\|MAUS_027559 | 5 | 324 | carbonic anhydrase 3 | 1 |
| 7 | P000026934\|10089..37671\|X179615\|MAUS_039323 | 4 | 269 | insulin-like growth factor-binding protein 2 precursor | 2 |
| 8 | P000025562\|10126..15853\|X171365\|MAUS_011752 | 4 | 245 | phosphoglycerate mutase 1, unnamed protein product | 1 |
| 9 | P000023042\|10105..21577\|X156567\|MAUS_019179 | 3 | 359 | malate dehydrogenase, mitochondrial precursor | 1 |
| 10 | P000018256\|10051..19446\|X127751\|MAUS_026701 | 3 | 214 | peroxiredoxin-6 | 1 |
| 11 | P000028443\|16065..25048\|X188757\|MAUS_028692 | 3 | 111 | alcohol dehydrogenase [NADP+] | 1 |
| 12 | P000052788\|11093..11751\|X133760\|MAUS_062929 | 2 | 165 | cofilin-2 | 2 |
| 13 | P000020601\|10034..14008\|X142106\|MAUS_031807 | 2 | 103 | 6-phosphogluconolactonase | 2 |
| 14 | P000026451\|11180..12320\|X176611\|MAUS_025132 | 2 | 86 | rho GDP-dissociation inhibitor 1 | 2 |
| 15 | P000025619\|10186..29074\|X171634\|MAUS_007656 | 2 | 79 | cAMP-regulated phosphoprotein 19 isoform 1 | 1 |
| 16 | P000024559\|10621..14464\|X165482\|MAUS_005161 | 2 | 60 | peroxiredoxin-2 | 1 |
| 17 | P000026744\|10033..11265\|X178434\|MAUS_036504 | 2 | 225 | 14 kDa phosphohistidine phosphatase | 1 |
| 18 | P000027044\|11348..15940\|X180333\|MAUS_031134 | 2 | 207 | RNA binding motif protein, X-linked | 1 |
| 19 | P000019273\|11820..23658\|X134237\|MAUS_028691 | 2 | 182 | peroxiredoxin-1 | 2 |
| 20 | P000020729\|12290..20903\|X143030\|MAUS_071644 | 2 | 170 | Eef1g protein | 1 |
| 21 | P000052151\|12644..14518\|X184127\|MAUS_041070 | 2 | 168 | eukaryotic translation initiation factor 5A, isoform CRA_j | 1 |
| 22 | P000022127\|20166..42003\|X151103\|MAUS_019961 | 2 | 160 | lamina-associated polypeptide 2 isoform delta | 1 |
| 23 | P000028168\|10167..37010\|X186979\|MAUS_021218 | 2 | 147 | guanosine diphosphate (GDP) dissociation inhibitor 2, isoform CRA_b and c | 1 |
| 24 | P000020999\|10119..26981\|X144660\|MAUS_036371 | 2 | 131 | plasminogen activator inhibitor 1 RNA-binding protein isoform 3 | 1 |
| 25 | P000022070\|10481..18563\|X150812\|MAUS_026728 | 2 | 120 | vimentin, unnamed protein | 1 |
| 26 | P000026522\|10089..36769\|X177075\|MAUS_028567 | 2 | 107 | thioredoxin domain-containing protein 12 precursor | 1 |
| 27 | P000020979\|13650..14999\|X144561\|MAUS_045427 | 2 | 104 | heterogeneous nuclear ribonucleoprotein H2 | 1 |

**Table S2: MALDI-TOF analysis from CM after heating at 60°C.** Proteins with a total ion score > 45 are listed. Data are given from one experiment with two runs.

|  | **Accession Number** | **Peptide Count** | **Total Ion Score** | **Blast** |
| --- | --- | --- | --- | --- |
| 1 | P000022070\|10481..18563\|X150812\|MAUS_026728 | 24 | 2142 | vimentin, unnamed protein |
| 2 | P000025352\|10111..18938\|X170045\|MAUS_004891 | 13 | 1494 | nestin |
| 3 | P000021382\|7754..13998\|X146990\|MAUS_004980 | 13 | 1299 | heterogeneous nuclear ribonucleoproteins A2/B1 isoform 2 |
| 4 | P000018018\|10907..19558\|X126344\|MAUS_025980 | 5 | 746 | 60 kDa heat shock protein, mitochondrial |
| 6 | P000021080\|10090..41734\|X145184\|MAUS_020849 | 5 | 487 | unnamed protein product |
| 5 | P000049063\|10208..11951\|X127700\|MAUS_017390 | 4 | 511 | fructose-bisphosphate aldolase C |
| 7 | P000019273\|11820..23658\|X134237\|MAUS_028691 | 4 | 435 | peroxiredoxin-1 |
| 8 | P000051665\|14256..15439\|X186510\|MAUS_034994 | 4 | 402 | Eef2 protein |
| 9 | P000025266\|10359..12260\|X169662\|MAUS_059970 | 4 | 373 | heat shock-related 70 kDa protein 2 |
| 10 | P000022358\|12325..18862\|X152492\|MAUS_037601 | 3 | 352 | nucleoside diphosphate kinase A |
| 11 | P000024559\|10621..14464\|X165482\|MAUS_005161 | 3 | 347 | peroxiredoxin-2 |
| 12 | P000049887\|10141..13142\|X127442\|MAUS_023456 | 3 | 304 | triosephosphate isomerase |
| 16 | P000026091\|10145..33559\|X174409\|MAUS_051391 | 3 | 189 | 14-3-3 protein gamma |
| 24 | P000020999\|10119..26981\|X144660\|MAUS_036371 | 3 | 162 | plasminogen activator inhibitor 1 RNA-binding protein isoform 3 |
| 13 | P000052150\|14007..14518\|X184135\|MAUS_041070 | 2 | 268 | eukaryotic translation initiation factor 5A, isoform CRA_j |
| 14 | P000026451\|11180..12320\|X176611\|MAUS_025132 | 2 | 218 | rho GDP-dissociation inhibitor 1 |
| 15 | P000050901\|31261..37899\|X160534\|MAUS_029309 | 2 | 212 | SPARC-like protein 1 precursor |
| 19 | P000023849\|138851..197346\|X161353\|MAUS_015222 | 2 | 167 | microtubule-associated protein 2 isoform 1 |
| 20 | P000018561\|11055..12743\|X129678\|MAUS_031972 | 2 | 167 | actin |
| 21 | P000018609\|10131..29603\|X129995\|MAUS_032366 | 2 | 164 | tropomyosin alpha-1 chain isoform 5 |
| 22 | P000050192\|10767..14471\|X158955\|MAUS_025381 | 2 | 164 | protein canopy homolog 2 precursor |
| 23 | P000024794\|10233..14024\|X166888\|MAUS_026864 | 2 | 163 | 78 kDa glucose-regulated protein precursor |
| 25 | P000023351\|10064..25998\|X158430\|MAUS_024359 | 2 | 162 | stress-70 protein, mitochondrial |
| 26 | P000052885\|10071..13417\|X174586\|MAUS_068220 | 2 | 154 | galectin-1 |
| 31 | P000020601\|10034..14008\|X142106\|MAUS_031807 | 2 | 111 | 6-phosphogluconolactonase |
| 40 | P000020539\|15102..16662\|X141828\|MAUS_026547 | 2 | 66 | transgelin-2 |
| 41 | P000022244\|31246..60587\|X151691\|MAUS_019894 | 2 | 66 | sodium-dependent neutral amino acid transporter B(0)AT2 |

**Table S3: MADLI-TOF analysis from heated VM CM.** Proteins with a total ion score > 45 and at least 2 protein counts are listed. n = number of experiments containing the named protein out of two independent experiments.

|  | **Accession Number** | **Peptide Count** | **Total Ion Score** | **pubmed** | **n** |
| --- | --- | --- | --- | --- | --- |
| 1 | ENSP00000446007 | 33 | 3128 | vimentin | 2 |
| 2 | ENSP00000357206 | 14 | 1113 | nestin | 2 |
| 3 | ENSP00000227378 | 11 | 718 | heat shock 70kDa protein 8 | 1 |
| 4 | ENSP00000419425 | 10 | 754 | [peptidyl-prolyl cis-trans isomerase A](http://www.ncbi.nlm.nih.gov/blast/Blast.cgi#alnHdr_10863927) | 1 |
| 5 | ENSP00000299198 | 9 | 626 | [creatine kinase](http://www.ncbi.nlm.nih.gov/blast/Blast.cgi#alnHdr_180570) | 1 |
| 6 | ENSP00000349748 | 9 | 627 | [splicing factor proline/glutamine-rich (polypyrimidine tract binding protein associated), isoform CRA_e](http://www.ncbi.nlm.nih.gov/blast/Blast.cgi#alnHdr_119627830) | 2 |
| 7 | ENSP00000380067 | 7 | 749 | Glycerinaldehyd-3-phosphat isoform 1 und 2 | 1 |
| 8 | ENSP00000384695 | 7 | 531 | [unnamed protein product](http://www.ncbi.nlm.nih.gov/blast/Blast.cgi#alnHdr_194391112) | 1 |
| 9 | ENSP00000405455 | 6 | 427 | [transketolase isoform 1](http://www.ncbi.nlm.nih.gov/blast/Blast.cgi#alnHdr_4507521) | 1 |
| 10 | ENSP00000422784 | 6 | 434 | albumin | 1 |
| 11 | ENSP00000261366 | 5 | 314 | [Lamin B1](http://www.ncbi.nlm.nih.gov/blast/Blast.cgi#alnHdr_15126742) | 1 |
| 12 | ENSP00000301522 | 5 | 565 | peroxiredoxin 2 | 2 |
| 13 | ENSP00000349101 | 5 | 528 | [heterogeneous nuclear ribonucleoprotein A2/B1, isoform CRA_a](http://www.ncbi.nlm.nih.gov/blast/Blast.cgi#alnHdr_410058693) | 2 |
| 14 | ENSP00000349960 | 5 | 368 | actin, beta | 1 |
| 15 | ENSP00000351777 | 5 | 385 | valosin containing protein | 1 |
| 16 | ENSP00000361152 | 5 | 490 | peroxiredoxin 1 | 2 |
| 17 | ENSP00000373620 | 5 | 495 | [60 kDa heat shock protein, mitochondrial](http://www.ncbi.nlm.nih.gov/blast/Blast.cgi#alnHdr_31542947) | 1 |
| 18 | ENSP00000290378 | 4 | 296 | actin, alpha, cardiac muscle 1 | 2 |
| 19 | ENSP00000360609 | 4 | 280 | heat shock protein 90kDa alpha (cytosolic), class B member 1 | 1 |
| 20 | ENSP00000361626 | 4 | 463 | [DNA-binding protein B, partial](http://www.ncbi.nlm.nih.gov/blast/Blast.cgi#alnHdr_181486) | 1 |
| 21 | ENSP00000374645 | 4 | 405 | [Chain A, A4v Mutant Of Human Sod1](http://www.ncbi.nlm.nih.gov/blast/Blast.cgi#alnHdr_47169370) | 1 |
| 22 | ENSP00000233893 | 3 | 187 | [10 kDa heat shock protein, mitochondrial](http://www.ncbi.nlm.nih.gov/blast/Blast.cgi#alnHdr_4504523) | 1 |
| 23 | ENSP00000234590 | 3 | 262 | enolase 1, (alpha) | 2 |
| 24 | ENSP00000253408 | 3 | 234 | glial fibrillary acidic protein | 1 |
| 25 | ENSP00000310219 | 3 | 206 | heat shock 70kDa protein 6 (HSP70B') | 1 |
| 26 | ENSP00000341289 | 3 | 226 | tubulin, beta 4B class IVb | 1 |
| 27 | ENSP00000341826 | 3 | 300 | heterogeneous nuclear ribonucleoprotein A1 | 1 |
| 28 | ENSP00000343282 | 3 | 224 | histone cluster 1, H4d | 1 |
| 29 | ENSP00000343690 | 3 | 203 | dihydropyrimidinase-like 3 | 1 |
| 30 | ENSP00000351777 | 3 | 163 | [transitional endoplasmic reticulum ATPase](http://www.ncbi.nlm.nih.gov/blast/Blast.cgi#alnHdr_6005942) | 1 |
| 31 | ENSP00000353552 | 3 | 187 | heterogeneous nuclear ribonucleoprotein K | 2 |
| 32 | ENSP00000357076 | 3 | 277 | transgelin 2 | 2 |
| 33 | ENSP00000360029 | 3 | 240 | SERPINE1 mRNA binding protein 1 | 2 |
| 34 | ENSP00000366708 | 3 | 238 | parkinson protein 7 | 2 |
| 35 | ENSP00000374645 | 3 | 382 | superoxide dismutase 1, soluble | 1 |
| 36 | ENSP00000377681 | 3 | 181 | GTPase activating protein (SH3 domain) binding protein 1 | 2 |
| 37 | ENSP00000391672 | 3 | 228 | tubulin, beta class I | 1 |
| 38 | ENSP00000416706 | 3 | 258 | actin, beta-like 2 | 1 |
| 39 | ENSP00000216181 | 2 | 77 | [myosin-9 [Homo sapiens]](http://www.ncbi.nlm.nih.gov/blast/Blast.cgi#alnHdr_12667788) | 1 |
| 40 | ENSP00000221166 | 2 | 98 | unnamed protein, neurofilament medium polypeptide isoform 1 | 1 |
| 41 | ENSP00000259791 | 2 | 180 | histone cluster 1, H2ab | 1 |
| 42 | ENSP00000301072 | 2 | 215 | tubulin, alpha 1c | 2 |
| 43 | ENSP00000307940 | 2 | 134 | eukaryotic translation elongation factor 2 | 1 |
| 44 | ENSP00000324422 | 2 | 90 | [zyxin, isoform CRA_a [Homo sapiens]](http://www.ncbi.nlm.nih.gov/blast/Blast.cgi#alnHdr_119572233) | 1 |
| 45 | ENSP00000331127 | 2 | 145 | [BolA-like protein 2 [Homo sapiens]](http://www.ncbi.nlm.nih.gov/blast/Blast.cgi#alnHdr_73622130) | 1 |
| 46 | ENSP00000333504 | 2 | 209 | [Chain A, Up1, The Two Rna-Recognition Motif Domain Of Hnrnp A1](http://www.ncbi.nlm.nih.gov/blast/Blast.cgi#alnHdr_20664272) | 1 |
| 47 | ENSP00000337060 | 2 | 100 | [nucleoside diphosphate kinase A isoform a](http://www.ncbi.nlm.nih.gov/blast/Blast.cgi#alnHdr_38045913) | 1 |
| 48 | ENSP00000339053 | 2 | 91 | eukaryotic translation elongation factor 1 alpha 1 | 1 |
| 49 | ENSP00000342026 | 2 | 213 | peroxiredoxin 6 | 1 |
| 50 | ENSP00000353408 | 2 | 137 | moesin | 1 |
| 51 | ENSP00000357280 | 2 | 168 | [lamin isoform C](http://www.ncbi.nlm.nih.gov/blast/Blast.cgi#alnHdr_5031875) | 1 |
| 52 | ENSP00000357340 | 2 | 101 | farnesyl diphosphate synthase | 1 |
| 53 | ENSP00000357429 | 2 | 204 | [fatty acid-binding protein, brain](http://www.ncbi.nlm.nih.gov/blast/Blast.cgi#alnHdr_4557585) | 1 |
| 54 | ENSP00000366568 | 2 | 185 | ubiquitin-like modifier activating enzyme 1 | 1 |
| 55 | ENSP00000369736 | 2 | 115 | [tubulin-specific chaperone A [Homo sapiens](http://www.ncbi.nlm.nih.gov/blast/Blast.cgi#alnHdr_4759212) | 1 |
| 56 | ENSP00000376037 | 2 | 110 | cytochrome c oxidase subunit VIb polypeptide 1 (ubiquitous) | 1 |
| 57 | ENSP00000376164 | 2 | 127 | lamin A/C | 1 |
| 58 | ENSP00000377298 | 2 | 83 | [nuclear mitotic apparatus protein 1](http://www.ncbi.nlm.nih.gov/blast/Blast.cgi#alnHdr_71361682) | 1 |
| 59 | ENSP00000378661 | 2 | 150 | aldolase A, fructose-bisphosphate | 2 |
| 60 | ENSP00000379933 | 2 | 180 | [triosephosphate isomerase isoform 1](http://www.ncbi.nlm.nih.gov/blast/Blast.cgi#alnHdr_4507645) | 1 |
| 61 | ENSP00000381216 | 2 | 163 | KH-type splicing regulatory protein | 1 |
| 62 | ENSP00000384925 | 2 | 160 | [RAN binding protein 1 variant](http://www.ncbi.nlm.nih.gov/blast/Blast.cgi#alnHdr_62898744) | 1 |
| 63 | ENSP00000385834 | 2 | 109 | [Chain A, Bismuth Bound Human Serum Transferrin](http://www.ncbi.nlm.nih.gov/blast/Blast.cgi#alnHdr_433286789) | 1 |
| 64 | ENSP00000386690 | 2 | 150 | Unknown protein | 1 |
| 65 | ENSP00000393151 | 2 | 142 | heterogeneous nuclear ribonucleoprotein U (scaffold attachment factor A) | 2 |
| 66 | ENSP00000400688 | 2 | 188 | heterogeneous nuclear ribonucleoprotein A3 | 2 |
| 67 | ENSP00000403962 | 2 | 139 | [glial fibrillary acidic protein isoform 2](http://www.ncbi.nlm.nih.gov/blast/Blast.cgi#alnHdr_196115290) | 1 |
| 68 | ENSP00000404372 | 2 | 124 | [heat shock cognate 71 kDa protein isoform 2](http://www.ncbi.nlm.nih.gov/blast/Blast.cgi#alnHdr_24234686) | 1 |
| 69 | ENSP00000405975 | 2 | 98 | peptidylprolyl isomerase A (cyclophilin A) | 1 |
| 70 | ENSP00000421592 | 2 | 189 | Aly/REF export factor | 2 |
| 71 | ENSP00000440127 | 2 | 112 | [unnamed protein product](http://www.ncbi.nlm.nih.gov/blast/Blast.cgi#alnHdr_194388798) | 1 |
| 72 | ENSP00000450627 | 2 | 136 | [thymopoietin isoform beta](http://www.ncbi.nlm.nih.gov/blast/Blast.cgi#alnHdr_73760405) | 1 |

**Table S4: MADLI-TOF analysis from heated C17.2 CM.** Proteins with a total ion score > 45 are listed. n = number of experiments containing the named protein out of two independent experiments.

|  | **Accession Number** | **Peptide Count** | **Total Ion Score** | **pubmed** | **n** |
| --- | --- | --- | --- | --- | --- |
| 1 | IPI:IPI00329872.1 | 48 | 4340 | collagen alpha-1(I) chain precursor [Mus musculus] | 2 |
| 2 | IPI:IPI00129571.3 | 21 | 1928 | Col3a1 protein | 2 |
| 3 | IPI:IPI00121120.3 | 19 | 1295 | Col5a2 protein | 2 |
| 4 | IPI:IPI00112904.1 | 16 | 1386 | 72 kDa type IV collagenase precursor | 2 |
| 5 | IPI:IPI00515360.8 | 15 | 887 | basement membrane-specific heparan sulfate proteoglycan core protein precursor [Mus musculus] | 2 |
| 6 | IPI:IPI00975193.1 | 13 | 843 | unnamed protein, fibronectin isoform b precursor | 2 |
| 7 | IPI:IPI00123744.1 | 11 | 937 | [cystatin-C precursor](http://www.ncbi.nlm.nih.gov/blast/Blast.cgi#alnHdr_31981822) | 1 |
| 8 | IPI:IPI00554989.3 | 8 | 580 | unnamed protein, peptidyl-prolyl cis-trans isomerase A | 1 |
| 9 | IPI:IPI00987580.1 | 8 | 540 | [PREDICTED: peptidyl-prolyl cis-trans isomerase A-like](http://www.ncbi.nlm.nih.gov/blast/Blast.cgi#alnHdr_407261558) | 1 |
| 10 | IPI:IPI00129903.2 | 7 | 471 | nidogen-2 precursor [Mus musculus] | 1 |
| 11 | IPI:IPI00126343.1 | 6 | 332 | SPARC | 1 |
| 12 | IPI:IPI00128689.2 | 5 | 316 | collagen alpha-1(V) chain precursor [Mus musculus] | 2 |
| 13 | IPI:IPI00131695.3 | 5 | 321 | serum albumin precursor [Mus musculus] | 2 |
| 14 | IPI:IPI00229517.5 | 5 | 407 | [galectin-1](http://www.ncbi.nlm.nih.gov/blast/Blast.cgi#alnHdr_6678682) | 2 |
| 15 | IPI:IPI00323357.3 | 5 | 244 | heat shock cognate 71 kDa protein | 1 |
| 16 | IPI:IPI00622847.2 | 5 | 372 | isoform 2 of heterogenous nuclear ribonucleoproteins A2/B1 | 1 |
| 17 | IPI:IPI00650029.1 | 5 | 422 | [SPARC precursor](http://www.ncbi.nlm.nih.gov/blast/Blast.cgi#alnHdr_6678077) | 1 |
| 18 | IPI:IPI00918214.1 | 5 | 424 | [clustrin](http://www.ncbi.nlm.nih.gov/blast/Blast.cgi#alnHdr_192150) | 1 |
| 19 | IPI:IPI00988539.1 | 5 | 304 | Vim, Uncharacterized protein | 2 |
| 20 | IPI:IPI00320420.3 | 4 | 320 | Clusterin | 1 |
| 21 | IPI:IPI00622235.5 | 4 | 201 | transitional endoplasmatic reticulum ATPase | 1 |
| 22 | IPI:IPI00649059.1 | 4 | 223 | Timp2, metalloproteinase inhibitor 2 precursor | 1 |
| 23 | IPI:IPI00986119.1 | 4 | 330 | [PREDICTED: actin, cytoplasmic 1-like isoform 4](http://www.ncbi.nlm.nih.gov/blast/Blast.cgi#alnHdr_345305332) | 2 |
| 24 | IPI:IPI00987541.1 | 4 | 280 | Aldoa, Fructose-bisphosphate aldolase A | 1 |
| 25 | IPI:IPI00129430.1 | 3 | 169 | splicing factor, proline- and glutamine-rich | 1 |
| 26 | IPI:IPI00317794.5 | 3 | 209 | nucleolin | 1 |
| 27 | IPI:IPI00319992.1 | 3 | 151 | 78 kDa glucose-regulated protein | 1 |
| 28 | IPI:IPI00621255.2 | 3 | 209 | Col2a1, Isoform 3 of Collagen alpha-1(II) chain | 1 |
| 29 | IPI:IPI00621255.2 | 3 | 188 | isoform 3 of collagen alpha-1(II) chain | 1 |
| 30 | IPI:IPI00648105.1 | 3 | 254 | Peroxiredoxin-1 | 1 |
| 31 | IPI:IPI00830331.1 | 3 | 171 | Pcolce, Uncharacterized protein | 1 |
| 32 | IPI:IPI00109588.4 | 2 | 111 | Col4a1, Collagen alpha-1(IV) chain | 2 |
| 33 | IPI:IPI00112487.1 | 2 | 87 | Igfbp4, Insulin-like growth factor-binding protein 4 | 1 |
| 34 | IPI:IPI00120848.1 | 2 | 106 | Mimecan | 1 |
| 35 | IPI:IPI00122272.2 | 2 | 89 | isoform long of extracellular matrix protein 1 | 1 |
| 36 | IPI:IPI00123181.4 | 2 | 118 | MYOSIN-9 | 1 |
| 37 | IPI:IPI00125778.4 | 2 | 137 | Transgelin-2 | 1 |
| 38 | IPI:IPI00128040.2 | 2 | 107 | Htra1, Serine protease HTRA1 | 2 |
| 39 | IPI:IPI00130589.8 | 2 | 149 | superoxide dismutase | 1 |
| 40 | IPI:IPI00133208.3 | 2 | 100 | heat shocl 10 kDa protein 1-like | 1 |
| 41 | IPI:IPI00221402.7 | 2 | 121 | Aldoa, Fructose-bisphosphate aldolase A | 2 |
| 42 | IPI:IPI00221463.3 | 2 | 107 | histone H2A type 3 | 1 |
| 43 | IPI:IPI00221528.1 | 2 | 164 | Actbl2, Beta-actin-like protein 2 | 1 |
| 44 | IPI:IPI00230616.2 | 2 | 91 | isoform 3 of collagen alpha-1(XVIII) chain | 1 |
| 45 | IPI:IPI00338452.3 | 2 | 223 | collagen alpha-2(IV) chain | 1 |
| 46 | IPI:IPI00400300.1 | 2 | 124 | isoform C of prelamin-A/C | 1 |
| 47 | IPI:IPI00420951.7 | 2 | 116 | [RecName: Full=Metalloproteinase inhibitor 2;](http://www.ncbi.nlm.nih.gov/blast/Blast.cgi#alnHdr_267133) | 1 |
| 48 | IPI:IPI00465574.2 | 2 | 73 | [unnamed protein product](http://www.ncbi.nlm.nih.gov/blast/Blast.cgi#alnHdr_74225344) | 1 |
| 49 | IPI:IPI00469541.1 | 2 | 101 | bone morphogenetic protein 1 precursor | 1 |
| 50 | IPI:IPI00515654.2 | 2 | 176 | elongation factor 1-delta isoform B | 1 |
| 51 | IPI:IPI00647981.3 | 2 | 212 | Ybx1, Y box protein 1 | 1 |
| 52 | IPI:IPI00830331.1 | 2 | 96 | unnamed protein product, procollagen C-endopeptidase enhancer 1 precursor | 1 |
| 53 | IPI:IPI00845840.1 | 2 | 110 | isoform M1 of pyruvate kinase isoenzymes M1/M2 | 1 |
| 54 | IPI:IPI00875420.1 | 2 | 75 | unnamed protein, PREDICTED: heterogeneous nuclear ribonucleoprotein K-like isoform 4 | 1 |
| 55 | IPI:IPI00114733.2 | 1 | 57 | SERPIN H1 | 1 |
| 56 | IPI:IPI00124707.2 | 1 | 72 | Fstl1, Follistatin-related protein 1 | 1 |
| 57 | IPI:IPI00127811.2 | 1 | 48 | Fractalkine | 1 |
| 58 | IPI:IPI00132314.1 | 1 | 69 | Nucleobindin-1 | 1 |
| 59 | IPI:IPI00132705.1 | 1 | 52 | myosin regulatory light chain 12B | 1 |
| 60 | IPI:IPI00137336.1 | 1 | 71 | Glypican-1 | 1 |
| 61 | IPI:IPI00137730.7 | 1 | 159 | Pebp1, Phosphatidylethanolamine-binding protein 1 | 1 |
| 62 | IPI:IPI00223770.2 | 1 | 45 | isoform 12 of CD44 antigen | 1 |
| 63 | IPI:IPI00226993.5 | 1 | 82 | Txn1, Thioredoxin | 1 |
| 64 | IPI:IPI00229812.1 | 1 | 79 | isoform 2 of macrophage colony-stimulating factor 1 | 1 |
| 65 | IPI:IPI00230427.5 | 1 | 53 | macrophage migration inhibitory factor | 1 |
| 66 | IPI:IPI00230494.6 | 1 | 54 | amyloid beta A4 protein isoform 3 precursor | 1 |
| 67 | IPI:IPI00322312.3 | 1 | 57 | rho GDP-dissociation inhibitor 1 | 1 |
| 68 | IPI:IPI00331286.3 | 1 | 100 | B2m, beta-2-microglobulin precursor | 1 |
| 69 | IPI:IPI00336324.11 | 1 | 54 | malate dehydrogenase, cytoplasmatic | 1 |
| 70 | IPI:IPI00353563.4 | 1 | 49 | fascin | 1 |
| 71 | IPI:IPI00399958.3 | 1 | 46 | calumenin isoform 2 | 1 |
| 72 | IPI:IPI00409817.2 | 1 | 47 | isoform non-muscle of myosin light polypeptide 6 | 1 |
| 73 | IPI:IPI00411146.5 | 1 | 55 | Lrp4, Isoform 2 of Low-density lipoprotein receptor-related protein 4 | 1 |
| 74 | IPI:IPI00457898.3 | 1 | 65 | phosphoglycerate mutase 1 | 1 |
| 75 | IPI:IPI00466069.3 | 1 | 58 | elongation factor 2 | 1 |
| 76 | IPI:IPI00467635.3 | 1 | 69 | isoform 2 of far upstream element binding protein 1 | 1 |
| 77 | IPI:IPI00473227.1 | 1 | 138 | Eno1, Enolase | 1 |
| 78 | IPI:IPI00515155.1 | 1 | 55 | [PREDICTED: nucleophosmin isoform 2](http://www.ncbi.nlm.nih.gov/blast/Blast.cgi#alnHdr_403290172) | 1 |
| 79 | IPI:IPI00553777.2 | 1 | 71 | Hnrnpa1, Putative uncharacterized protein | 1 |
| 80 | IPI:IPI00648764.1 | 1 | 51 | unnamed protein, protein DJ-1 | 1 |
| 90 | IPI:IPI00649216.1 | 1 | 115 | isoform 2 of calsyntenin-1 (fragment) | 1 |
| 91 | IPI:IPI00650039.1 | 1 | 46 | profilin | 1 |
| 92 | IPI:IPI00652811.2 | 1 | 52 | unnamed protein, annexin A1 | 1 |
| 93 | IPI:IPI00751369.1 | 1 | 53 | L-lactate dehydrogenase A chain isoform 2 | 1 |
| 94 | IPI:IPI00758024.1 | 1 | 77 | Prdx6, Uncharacterized protein | 1 |
| 95 | IPI:IPI00758024.1 | 1 | 114 | Peroxiredoxin-6 | 1 |
| 96 | IPI:IPI00762091.4 | 1 | 68 | isoform 1 of C-type mannose receptor 2 | 1 |
| 97 | IPI:IPI00817004.1 | 1 | 119 | isoform long of heterogenous nuclear ribonucleoprotein A1 | 1 |
| 98 | IPI:IPI00828909.4 | 1 | 55 | [PREDICTED: peroxiredoxin-4 isoform X3](http://www.ncbi.nlm.nih.gov/blast/Blast.cgi#alnHdr_511899372) | 1 |
| 99 | IPI:IPI00831295.1 | 1 | 47 | 1500002O20Rik, Isoform 1 of Protein SMG9 | 1 |
| 100 | IPI:IPI00858118.1 | 1 | 59 | 8 kDa protein | 1 |
| 101 | IPI:IPI00876563.1 | 1 | 86 | unnamed protein, mCG22278 | 1 |
| 102 | IPI:IPI00928204.1 | 1 | 53 | sulfated glycoprotein 1 isoform C preproprotein | 1 |
| 103 | IPI:IPI00970121.1 | 1 | 112 | unnamed protein product, heterogeneous nuclear ribonucleoprotein U, isoform CRA_b | 1 |
| 104 | IPI:IPI00970203.1 | 1 | 53 | [PREDICTED: proteasome subunit alpha type-6 isoform X2](http://www.ncbi.nlm.nih.gov/blast/Blast.cgi#alnHdr_507945825) | 1 |
| 105 | IPI:IPI00986893.1 | 1 | 52 | Peroxiredoxin-2-like | 1 |
| 106 | IPI:IPI00988063.1 | 1 | 53 | triosephosphate isomerase | 1 |
| 107 | IPI:IPI00989404.1 | 1 | 49 | 60S ribosomal protein L12-like | 1 |

**Table S5:** **MALDI-TOF analysis of SNL-CM.** Proteins with a total ion score > 45 are listed. Data are given from one experiment with two runs.

|  | **Accession Number** | **Peptide Count** | **Total Ion Score** | **Blast** |
| --- | --- | --- | --- | --- |
| 1 | P000022070\|10481..18563\|X150812\|MAUS_026728 | 3 | 133 | vimentin |
| 2 | P000018561\|11055..12743\|X129678\|MAUS_031972 | 2 | 123 | actin, alpha skeletal muscle |
